# Single‐Nucleus Transcriptome Profiling of Locally Advanced Cervical Squamous Cell Cancer Identifies Neural‐Like Progenitor Program Associated with the Efficacy of Radiotherapy

**DOI:** 10.1002/advs.202300348

**Published:** 2023-07-09

**Authors:** Lei Zhang, Jun Ma, Di Zhou, Junjun Zhou, Bin Hu, Xiumei Ma, Jianming Tang, Yongrui Bai, Haiyan Chen, Ying Jing

**Affiliations:** ^1^ Department of Radiation Oncology Renji Hospital School of Medicine Shanghai Jiao Tong University Shanghai 200127 China; ^2^ Eye Institute Eye & ENT Hospital Shanghai Medical College Fudan University Shanghai 200031 China; ^3^ Department of Radiation Oncology The First Hospital of Lanzhou University Lanzhou University Lanzhou 730000 China; ^4^ Center for Intelligent Medicine Research Greater Bay Area Institute of Precision Medicine (Guangzhou) Fudan University Guangzhou 511458 China

**Keywords:** cancer‐associated fibroblasts, cervical squamous cell cancer, neural‐like progenitor, radiotherapy, single‐nucleus transcriptome

## Abstract

Radiotherapy is the first‐line treatment for locally advanced cervical squamous cell cancer (CSCC). However, ≈50% of patients fail to respond to therapy and, in some cases, tumors progress after radical radiotherapy. Here, single‐nucleus RNA‐seq is performed to construct high‐resolution molecular landscapes of various cell types in CSCC before and during radiotherapy, to better understand radiotherapy related molecular responses within tumor microenvironment. The results show that expression levels of a neural‐like progenitor (NRP) program in tumor cells are significantly higher after radiotherapy and these are enriched in the tumors of nonresponding patients. The enrichment of the NRP program in malignant cells from the tumors of nonresponders in an independent cohort analyzed by bulk RNA‐seq is validated. In addition, an analysis of The Cancer Genome Atlas dataset shows that NRP expression is associated with poor prognosis in CSCC patients. In vitro experiments on the CSCC cell line demonstrate that downregulation of neuregulin 1 (NRG1), a key gene from NRP program, is associated with decreased cell growth and increased sensitivity to radiation. Immunohistochemistry staining in cohort 3 validated key genes, NRG1 and immediate early response 3 from immunomodulatory program, as radiosensitivity regulators. The findings reveal that the expression of NRP in CSCC can be used to predict the efficacy of radiotherapy.

## Graphical abstract

About half of locally advanced cervical squamous cell cancer (CSCC) patients receiving radiotherapy experience radioresistance. This study reveals that the expression levels of a neural‐like progenitor (NRP) program in malignant cells are significantly elevated in the tumors from nonresponders or after radiotherapy, and higher NRP expression is associated with poor prognosis in CSCC patients.

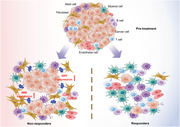

## 1 Introduction

Cervical cancer ranks the third leading cause of cancer deaths in women worldwide and it is persistently the second leading cause of cancer deaths in women aged 20–39 years.^[^ ^1^ ^]^ There are two main types of cervical cancers, and cervical squamous cell carcinoma (CSCC) accounts for almost 90% of cases.^[^ ^1b^ ^]^ Surgery, radiotherapy, and/or chemotherapy are frequently used in the clinical setting, as they are the most effective treatments for cervical cancer. Accumulating evidence has indicated that radiotherapy, which activates cytotoxic signaling pathways to promote cancer cell death, is generally used as the first‐line treatment for CSCC, especially in locally advanced cases.^[^ ^2^ ^]^ However, the treatment efficiency of radiotherapy is far less than expected and cervical cancer‐specific mortality remains high,^[^ ^3^ ^]^ with about half of locally advanced CSCC patients experiencing a recurrence after radiotherapy.^[^ ^4^ ^]^ Therefore, identifying the molecular markers and exploring the underpinning mechanisms of radiotherapy are necessary for improving the efficiency of this treatment in locally advanced CSCC.

It is widely accepted that high heterogeneity in both the tumor and its microenvironment (TME) allows a cancer to evolve continuously and this often leads to the failure of treatment regimens.^[^ ^5^ ^]^ Previous studies have shown that tumor heterogeneity,^[^ ^6^ ^]^ TME,^[^ ^7^ ^]^ the immune system,^[^ ^8^ ^]^ and metabolism states^[^ ^9^ ^]^ are considered as the significant factors affecting its sensitivity to radiotherapy. In addition, various cytoprotective‐related pathways, subsequently elicited by cytotoxic signaling pathways after exposure to radiotherapy,^[^ ^10^ ^]^ in both the tumor and TME component cells warrant further exploration. Consequently, dissecting the molecular dynamics of the different cell types and the remodeling of the TME in radiotherapy‐exposed CSCC tissues will help us to understand the fundamental mechanisms underlying these radiotherapy‐induced responses.

Currently, studies at the single‐cell level in cervical cancer are limited and these are largely focused on treatment‐naïve tumors to determine the changes that occur during cancer progression. Li et al. observed different metabolic states in tumor‐derived endothelial cells during cervical cancer progression when compared with normal endothelial cells.^[^ ^11^ ^]^ They also established the dynamic changes of TME during cervical cancer progression by single‐cell transcriptomic profiles of normal cervix, intraepithelial neoplasia, primary tumor, and metastatic lymph node tissues.^[^ ^12^ ^]^ Therefore, the evidence of radio‐sensitivity of CSCC at the single‐cell level is still lacking, primarily due to the difficulties in obtaining multiple tumor samples during treatment of patients. Single‐nucleus RNA sequencing (snRNA‐seq) has the unique power to profile archived frozen tissues accrued over time and thus this technique enables the longitudinal analysis of individual patients.^[^ ^13^ ^]^ In addition, snRNA‐seq can also chart a comprehensive tumor cell atlas as it is a dissociation‐free process^[^ ^14^ ^]^ and it allows the minimal disturbance of gene expression caused by enzyme digestion, which is required for single‐cell RNA‐seq.^[^ ^15^ ^]^

In this study, we collected banked paired pre‐ and on‐treatment CSCC specimens, which allowed for the rigorous analysis of treatment‐related changes. Next, we utilized snRNA‐seq to comprehensively characterize the temporal dynamics of the major cell types before and during radiotherapy, which allowed the elucidation of key factors related to the radiotherapy responses in locally advanced CSCC. We further collected an independent cohort and performed bulk RNA‐seq to validate the snRNA‐seq‐derived data, that is, increased expression of a neural‐like progenitor program (NRP) in tumor cells that was significantly associated with radiotherapy responses in locally advanced CSCC. These findings will allow us to explain the basic mechanisms of radiotherapy treatment. They may also provide potential predictive biomarkers of the radiotherapy responses as well as novel therapeutic targets that could be used to improve the response rate of patients with locally advanced CSCC to radiotherapy.

## 2 Results

### 2.1 SnRNA‐seq of Stored Frozen Human Cervical Tumors Before and During Radiotherapy

**Figure 1:**
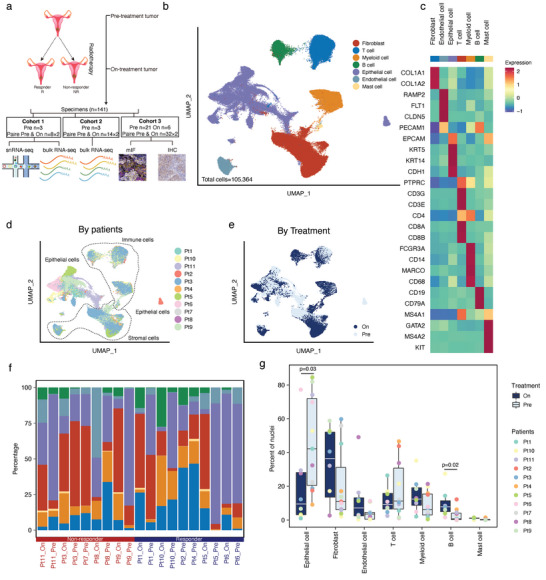
Analysis of the diverse cell types of pre‐ and on‐radiotherapy treatment cervical squamous cell carcinoma (CSCC) by snRNA‐seq. a) Experimental workflow of CSCC sample collection for snRNA‐seq and bulk RNA‐seq. b) Uniform manifold approximation and projection (UMAP) embedding of single‐nucleus profiles of pre‐ and on‐treatment tumors from all patients. The cells are colored according to their major cell types. c) A heatmap of the major cell type marker genes for each cell clusters (the color legend is shared with panel b). d,e) UMAP of nuclei captured across all tumors, colored according to the individual patient (d) and exposure to radiotherapy treatment (e). f) The cell composition in each tumor. The percentages (*y* axis) of the major cell clusters (the color legend is shared with panel b) across pre‐ and on‐treatment tumors. The color of the *x‐*axis text indicates the response status of patients. g) Alteration of tumor composition by radiotherapy treatment. The percentages (*y*‐axis) of each cell type (*x*‐axis) among all the nuclei. Comparisons were performed by using the two‐sided Wilcoxon rank‐sum test.

We collected 19 freshly frozen and histologically confirmed CSCC pre‐ and on‐radiotherapy specimens from 11 patients for snRNA‐seq (**Figure** **1**a, Experimental Section; and Table S1, Supporting Information). To better depict the temporal dynamic single‐cell transcriptomic reprogramming, we collected paired samples before and during radiotherapy from 8 patients (Figure 1a; and Table S1, Supporting Information). After removing the low‐quality snRNA‐seq nuclei, we obtained 105 364 high‐quality snRNA‐seq profiles that consisted of various cell types from all the samples (Figure 1b,d,e). We employed the Harmony algorithm to mitigate possible batch effects.^[^ ^16^ ^]^ Unsupervised clustering of single‐nucleus profiles identified seven major cell clusters, and we annotated these by their canonical marker genes (Figure 1c). Among the treatment subgroups (pre‐ and on‐treatment), the proportions of epithelial cells were significantly lower (*p*‐value = 0.03), while those of B cells were significantly higher (*p*‐value = 0.02) in the tumors after treatment (Figure 1f,g). Considering that the snRNA‐seq technique recovered more parenchymal cell types and less immune cells than single‐cell RNA sequencing,^[^ ^14a^ ^]^ we focused on non‐immune cells in our subsequent analysis.

### 2.2 Mapping the Molecular Changes of Malignant Cells During Radiotherapy

**Figure 2:**
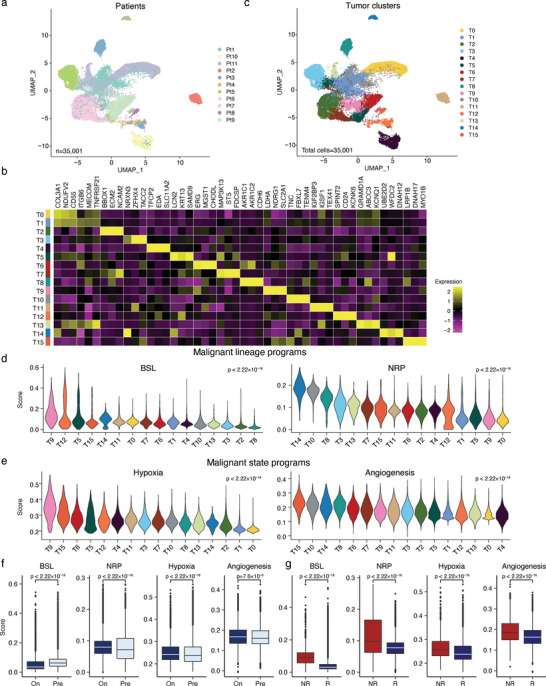
The malignant cell states are differentially remodeled by radiotherapy. a) UMAP of malignant cells captured across all tumors, colored according to the individual patient. b) A heatmap of the scaled normalized expression for the cell type marker genes determined by the two‐sided Wilcoxon rank‐sum test with Bonferroni FDR (false discovery rate) correction. c) UMAP of malignant cells captured across all tumors, colored according to the subclusters. d) Score distributions for the lineage programs, basaloid (BSL), and neural‐like progenitor (NRP), within each malignant cell subclusters (the color legend is shared with panel (c). e) Score distributions for state programs, hypoxia, and angiogenesis, within each malignant cell subclusters (the color legend is shared with panel (c). f,g) Significance of the differential program enrichment (*p*‐value) among subclusters was determined by using the Kruskal–Wallis test. Scores for BSL, NRP, hypoxia, and angiogenesis in the pre‐/on‐radiotherapy samples (f) and on‐treatment samples from the responders/nonresponders (g). Significance of differential program expression was determined by the two‐sided Wilcoxon rank‐sum test. Boxplots indicate the median ± 1 quartile, with whiskers extending from the hinge to the smallest and largest values within 1.5 interquartile range from the box boundaries. On: on‐treatment. Pre: pretreatment. NR: nonresponder. R: responder.

Next, we determined the malignant cells by the inferred copy‐number variations in epithelial cells (Figure S1, Supporting Information), which were comparable to those derived from The Cancer Genome Atlas (TCGA) cervical cancer cohort.^[^ ^17^ ^]^ Consistent with previous studies,^[^ ^16a,b^ ^]^ the malignant cells generally clustered according to the patient (**Figure** **2**a; and Figure S2a–c, Supporting Information), whereas immune and stromal cells from different patients were clustered together by cell types (Figure 1d). Ninety‐eight percent of the epithelial cells were malignant cells, and their proportion of the total was significantly lower in on‐treatment tumors (*p*‐value = 0.017; Figures S2d and S3a, Supporting Information). The 35001 malignant cells derived from all the samples clustered into 16 distinct subclusters (Figure 2b,c).

To determine whether any of malignant cell subclusters we recovered resembled different cell lineages, we scored individual cells according to published snRNA‐seq generated lineage programs (Figure S4 and Table S2, Supporting Information).^[^ ^16a^ ^]^ We found that the expression of basaloid (BSL) program, with enriched stemness‐relevant genes, was strongly elevated in a subset of cells within the T9, T12, and T5 subclusters. Conversely, the expression of NRP program, with enriched neuronal development/migration/adhesion relevant genes, was elevated in cells of the T14, T10, and T8 subclusters (Figure 2d; and Figure S3b, Supporting Information). We examined the expression of the unique neuroendocrine‐like (NEN) and NRP genes to rule out the possibility that the NRP and NEN programs overlapped. We observed that only the NRP genes were highly expressed in the T14, T10, and T8, and not the NEN genes (Figure S3c, Supporting Information). Next, we evaluated the expression of the hallmark gene sets from MSigDB^[^ ^18^ ^]^ to determine the cell states of the malignant cells. Cells from the T14, T10, and T8 subclusters were more highly expressed with respect to the angiogenesis pathway, which facilitates tumor growth,^[^ ^19^ ^]^ while cells from the T9, T12, and T5 subclusters had higher expression of genes associated with the hypoxia pathway, which is associated with radioresistance^[^ ^7^, ^20^ ^]^ (Figure 2e).

Having identified subsets of malignant cells that bear some features associated with tumor growth and radiotherapy sensitivity, we next examined the differences in gene expression based on exposure and response to the treatment. Radiotherapy was observed to be associated with significant differences in the expression of lineages and cell state programs (Figure 2f,g). The expression of malignant NRP (*p* < 2.2 × 10^−16^) program was significantly higher in on‐treatment versus pretreatment samples, whereas the expression of BSL (*p* < 2.2 × 10^−16^) program was lower (Figure 2f). However, the expression of hypoxia and angiogenesis program were both higher in on‐treatment samples (Figure 2f). Of note, four of the malignant programs, BSL, NRP, hypoxia, and angiogenesis, were highly expressed in malignant cells from on‐treatment tumors of nonresponders (Figure 2g), which suggested treatment‐sensitivity relevance of their programs in these cell types. Moreover, we performed spearman correlation analysis between expression levels of NRP program and all genes in malignant cells to find potential mechanisms involved in the increasing expression levels of NRP programs. Of note, the majority of the significantly NRP associated genes were positively correlated with NRP program expression (Figure S5a, Supporting Information). It is widely known that an important way of radiotherapy‐killing is to induce DNA damage and growth arrest of cancer cells.^[^ ^2^, ^21^ ^]^ We also observed that significantly correlated genes were highly enriched in DNA repair or mitotic cell cycle related pathways (Figure S5b, Supporting Information). Differential pathway expression analysis indicated elevated expression of cell cycle and cell apoptosis related pathways, including mitotic spindle, G2M checkpoint, and suppression of apoptosis, in NRP highly expressed (NRP‐high) malignant cells. Expression of transforming growth factor‐β (TGF‐β) signaling, a versatile factor that participates or regulates cell development, proliferation and differentiation, was also increased in NRP‐high cells (Figure S5c, Supporting Information). These results suggested that increased NRP expression in malignant cells after radiotherapy may be accompanied with elevated DNA repair ability and cellular mitosis, which could subsequently cause higher tumor cell growth ability.

### 2.3 Radiotherapy of Treatment‐ and Response‐Associated CAF Subpopulations

**Figure 3:**
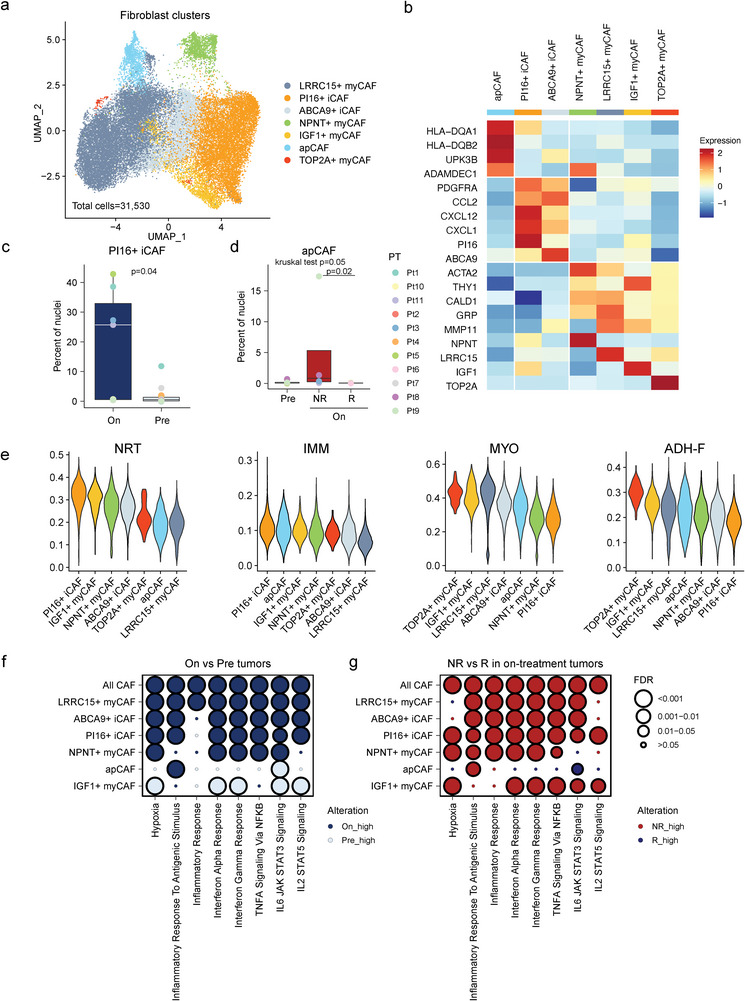
The distribution and cell states of CAFs that are associated with radiotherapy. a) UMAP projection of snRNA‐seq CAFs isolated from all samples. The cells were colored according to the subclusters. b) A heatmap of scaled normalized expression for cell type marker genes determined by the two‐sided Wilcoxon rank‐sum test with Bonferroni FDR (false discovery rate) correction. c,d) A comparison of PI16+ iCAF c) and apCAF d) proportion in different groups. Comparisons between pre‐ versus on‐treatment tumors and nonresponders (NR) versus responders (R) were conducted by the two‐sided Wilcoxon rank‐sum test. e) Score distributions for CAF programs, neurotropic (NRT), immunomodulatory (IMM), myofibroblastic progenitor (MYO), and adhesive fibroblast (ADH‐F), within each CAF subclusters (the color legend is shared with panel b). f,g) Significantly differential expression of hallmark gene sets in each CAF subclusters and all CAFs. The dot size indicates the FDR values. The color of the dots indicates the alteration between groups. Boxplots indicate the median ± 1 quartile, with whiskers extending from the hinge to the smallest and largest values within 1.5 interquartile range from the box boundaries. CAF: cancer associated fibroblast; TNFA: tumor necrosis factor; α; IL6: interleukin 6; IL2: interleukin 2; STAT3: signal transducers and activators of transcription 3; STAT5: signal transducers and activators of transcription 5; JAK: Janus kinase.

Fibroblasts, which are the major components of the TME, are reported to play heterogenous roles.^[^ ^22^ ^]^ These include promoting tumor proliferation^[^ ^23^ ^]^ as well as T cell exclusion from tumor nests.^[^ ^24^ ^]^ We recovered 31530 cancer‐associated fibroblasts (CAFs) from all samples, and unsupervised clustering revealed 7 different cell subclusters (**Figure** **3**a,b), including one antigen‐presenting CAF (apCAF), two inflammatory CAF (iCAF), and four myofibroblastic CAF (myCAF) subclusters. Interestingly, we observed an increased proportion of PI16+ iCAF (*p*‐value = 0.04) after radiotherapy treatment (Figure 3c) and decreased numbers of apCAF (*p*‐value = 0.02) in patients who responded better to radiotherapy treatment (Figure 3d). However, no significant differences were detected in the other five types of CAFs (Figure S6a,b, Supporting Information). We further calculated the expression of the four snRNA‐seq‐derived CAF programs.^[^ ^16a^ ^]^ The neurotropic (NRT), immunomodulatory (IMM), myofibroblastic progenitor (MYO), and adhesive fibroblast (ADH‐F) programs, which could be used to determine the functional phenotypes of these subclusters. PI16+ iCAF had the highest expression of NRT and IMM programs, and the lowest expression of MYO and ADH‐F programs (Figure 3e). ApCAF also had a relatively higher expression of IMM program (Figure 3e). Meanwhile, we observed significantly increased expression of interferon, TGF, and interleukin pathways in IMM highly expressed CAFs (Figure S6c, Supporting Information). Cell apoptosis, DNA damage, and hypoxia related pathways were also increased in IMM highly expressed cells. We then sought to explore whether the inflammation‐related transcriptional states were associated with radiotherapy treatment and efficacy. We found that the expression of inflammation‐related pathways, such as interferon, tumor necrosis factor and interleukin 6, were significantly higher in most CAF subclusters of the on‐treatment tumors when compared to those of pretreatment tumors (Figure 3f). Moreover, CAFs from the nonresponders’ on‐treatment tumors had a higher expression level of these pathways than tumors from responders (Figure 3g). Collectively, our observations of CAFs indicated that they had an inflammatory‐regulating role in the TME of cervical cancer and that they were associated with the efficacy of radiotherapy.

### 2.4 Ligand–Receptor Cell–Cell Interactions in the Context of Radiotherapy

**Figure 4:**
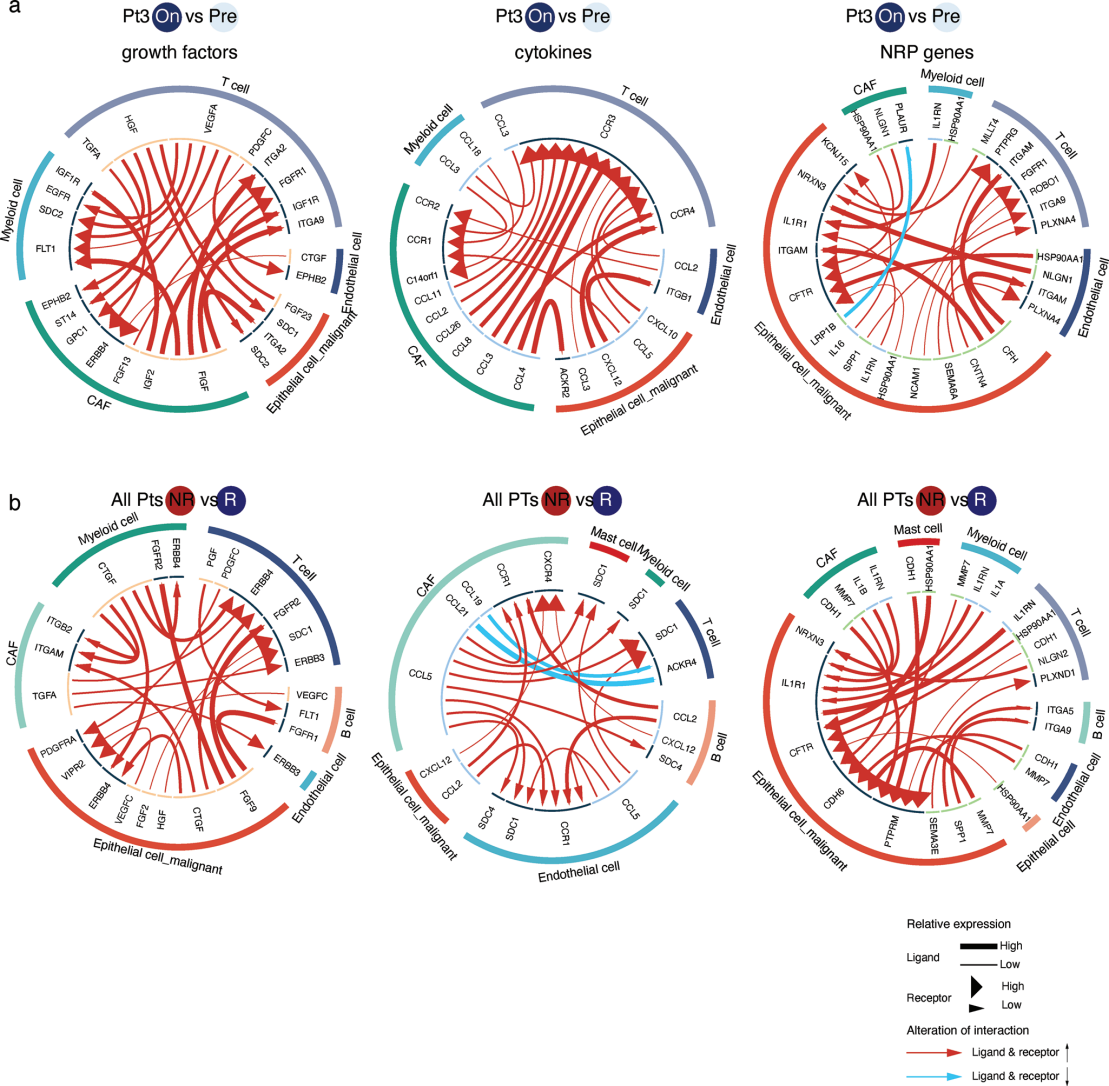
The enriched ligand–receptor cell–cell interactions across major cell types in radiotherapy‐exposed tumors and nonresponders. a,b) Representative circos plots showing details of the top 20 differentially regulated growth factor (left), cytokines (middle), and NRP genes (right) ligand–receptor pairs when comparing on‐ versus pretreatment samples in Pt3 a) and nonresponders versus responders b). NR: nonresponder. R: responder. The lines are colored according their alterations. The size of the lines indicates the relative expression of ligands. The size of the arrows indicates the relative expression of receptors.

Given the results of the malignant programs, such as the upregulation of NRP in malignant cells and the inflammation‐related pathway upregulation in CAFs from nonresponders, we hypothesized that different cell clusters may be involved in a complex cell–cell crosstalk. Therefore, we utilized iTALK^[^ ^16^, ^25^ ^]^ to identify signaling between malignant cells and other cell types based on iTALK's built‐in receptor–ligand pair databases. We identified altered cellular interactions with respect to growth factor, cytokine, and NRP genes that were significantly and differentially upregulated in on‐treatment versus its respective pretreatment samples that were obtained from individual patients (**Figure** **4**a; and Figure S7, Supporting Information). It is not possible to obtain both responsive and nonresponsive tumors in one patient, so we performed response‐related differential cell–cell interaction analysis on all patients. Consistent with the higher expression levels of the angiogenesis pathway observed in the on‐treatment tumors from NR, we observed increased numbers of crosstalk between the vascular endothelial growth factor receptor–ligand pairs in these tumors (Figure 4a,b). Among the cytokine–receptor pairs, CAFs showed a greater number of differentially upregulated communication pairs between the other cell types in the on‐treatment tumors from nonresponders (Figure 4a). Notably, we observed more tumor cells and other cell type interactions between ligands and receptors of NRP genes in the tumors during treatment when compared to tumors obtained from before treatment (Figure 4a). There were also more interactions of NRP genes in tumors of nonresponders compared to those of responders (Figure 4b). Taken together, the results suggest that the cytokine‐ and NRP‐related cell–cell interactions between CAFs and malignant cells may be involved in radiotherapy‐induced responses and subsequently these are likely to mediate treatment efficacy.

### 2.5 Increased Expression of NRP Program was Associated with Poor Outcomes

**Figure 5:**
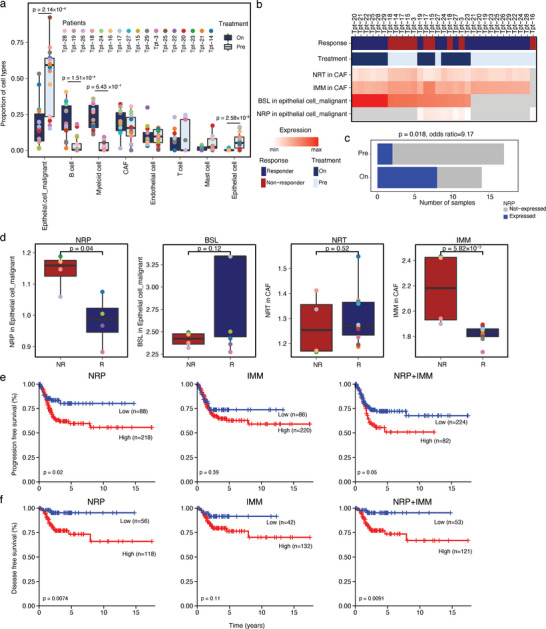
Bulk RNA‐seq analysis of a second independent cohort and TCGA dataset. a) Alteration of relative cell abundance deconvolved via CIBERSORTx according to the individual treatment status. The relative abundance (*y*‐axis) of each cell type (*x*‐axis) for all the nuclei. Comparisons were performed by using the two‐sided Wilcoxon rank‐sum test. b) A heatmap of the scaled normalized expression for NRT in CAF, IMM in CAF and BSL and NRP in the malignant cells as inferred by CIBERSORTx. c) The number (*x*‐axis) of samples (colored according to the NRP expression status) in the pre‐ and on‐treatment tumors (*y*‐axis). d) Comparisons of the program expression during radiotherapy treated tumors between nonresponders (NR) and responders (R). Boxplots indicate the median ± 1 quartile, with whiskers extending from the hinge to the smallest and largest values within 1.5 interquartile range from the box boundaries. e,f) The progression‐free survival (e) and disease‐free survival (f) of cases as stratified by the program expression levels. Statistical analyses were performed by using Kaplan–Meier analysis.

To validate our observations, we collected a second independent locally advanced CSCC patient cohort, that included pre‐ and on‐treatment tumors from 17 patients (14 of which had matched pre‐ and on‐treatment specimens, as described in Figure 1a; and Table S3, Supporting Information). We performed bulk RNA‐seq to fully capture their temporal transcriptomic landscape during radiotherapy. To test reliability of applying snRNA‐seq‐based expression patterns from bulk transcriptomic profiles, we performed bulk RNA‐seq on all the specimens collected, including those that had undergone snRNA‐seq. We deconvolved the bulk expression data with the snRNA‐seq cell‐type signatures by utilizing the CIBERSORTx algorithm.^[^ ^26^ ^]^ Next, we analyzed the correlation between the cell percentages obtained by using the snRNA‐seq data and deconvolved the cell abundance from the bulk RNA‐seq data. The results showed a significantly positive correlation between the snRNA‐seq‐ and bulk RNA‐seq‐derived results at both the patient and the cell‐type levels (Figure S8, Supporting Information). Therefore, we used CIBERSORTx to infer the cell abundance and expression of programs in the independent cohort. Similar as our snRNA‐seq results, we found that most of the cells were malignant cells, and the proportions of malignant (*p*‐value = 2.14 × 10^−4^) and B cells (*p*‐value = 1.51 × 10^−4^) were significantly altered in the tumors during radiotherapy (**Figure** **5**a).

We further calculated the expression of BSL and NRP programs in the malignant cells and in the NRT and IMM programs in CAFs (Figure 5b). The expression of NRP was successfully determined in only two pretreatment and eight on‐treatment tumors (Figure 5b), which indicated that radiotherapy was positively associated with the NRP program expression in malignant cells (*p‐*value = 0.02, odds ratio = 9.17; Figure 5c). For the comparison of expression levels among the treatment subgroups, due to the limited number of pretreatment samples expressing NRP, we only observed increased expression levels of IMM in CAFs in the on‐treatment tumors (Figure S9a, Supporting Information). Of note, the expression of NRP in malignant cells and IMM in CAFs was significantly higher in the tumors of nonresponders versus that of responders (*p*‐value = 0.04; *p*‐value = 5.82 × 10^−3^; Figure 5d). This finding validated the negative associations between the expression of the two programs, NRP and IMM, as well as the radiotherapy response we obtained by using snRNA‐seq. Unfortunately, although we collected two independent cohorts, the sample sizes were still limited. Hence, we took advantage of TCGA cervical cancer dataset to demonstrate the relationship between patient outcomes and the expression of NRP and IMM in a larger CSCC cohort. Kaplan–Meier analysis of the time to progression (TTP) and disease‐free survival (DFS) revealed that higher expression of NRP and NRP+IMM, but not IMM, was associated with a shorter TTP and DFS (Figure 5e,f). We also found that only expression of NRP was associated with overall survival (Figure S10, Supporting Information). These findings paralleled the results of another study, which also showed an association between the NRP program expression and worsening outcomes in pancreatic cancer.^[^ ^16a^ ^]^

### 2.6 Validation of Key Genes of NRP and IMM Program as Radiosensitivity Regulators

We next aimed to assess the biological functions relevant to radiotherapy of key genes in the NRP and IMM programs in radiotherapy. Among the 200 genes in NRP program, we found that 143 genes were significantly upregulated in malignant cells from nonresponders when compared with responders (Figure S11a, Supporting Information). To identify key genes in NRP program, we interrogated the gene essentialities of 143 upregulated genes for the proliferation and survival of cervical cancer cell lines by analyzing dependency scores from DepMap data portal ^[^ ^27^ ^]^ (Figure S11b, Supporting Information). Next, we analyzed the endogenous expression levels of the top 20 genes based on DepMap data in cervical cancer cell lines (Figure S11c, Supporting Information). Considering that functions of the top genes, such as hypoxia inducible factor 1 subunit α, are well‐characterized, we selected neuregulin 1 (NRG1) as our candidate gene in following functional experiments. NRG1 was reported to be involved in neural development,^[^ ^28^ ^]^ regulate diverse biological functions in several types of cells, such as differentiation, proliferation, and apoptosis,^[^ ^29^ ^]^ and be potentially related to tyrosine kinase inhibitors resistance in lung cancer.^[^ ^30^ ^]^

**Figure 6:**
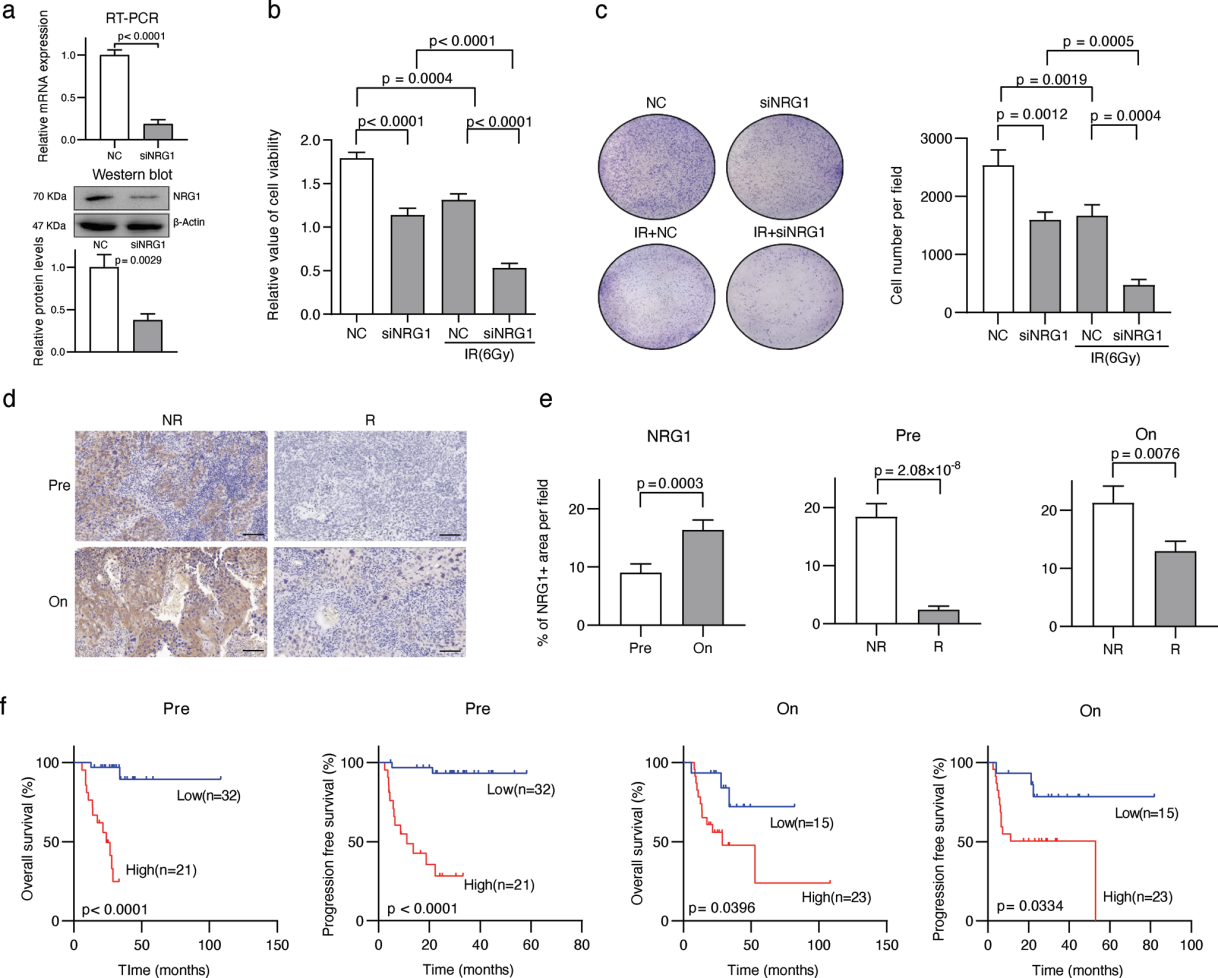
Validation of NRG1, a NRP key gene, as radiosensitivity regulators. a) Real‐time PCR and western blot were utilized to determine the knock down efficiency of NRG1 by small interfering RNA (siRNA) in SiHa cells. b) Cell viability results in different groups (*n* = 6) determined by Cell Counting Kit‐8 (CCK‐8) assay. After 24 h of NC or siNRG1 transfection, cells in indicated groups were exposed to 6 Gy IR. CCK‐8 assay was performed after 48 h of IR treatment. c) Crystal violet staining of cell colonies in different groups (*n* = 3). Transfection and IR treatment steps were similar as CCK‐8 assay. Cells were fixed and stained after 12 days of IR treatment, d) Representative immunohistochemistry images of NRG1 in CSCC specimens, scale bar = 100 µm. e) Comparison of NRG1 expression positivity in random selected fields. *p*‐value was calculated by using the two‐sided Wilcoxon rank‐sum test. f) Kaplan–Meier curve of overall and progression free survival of cases stratified by the expression of NRG1. RT‐PCR: real‐time PCR; IR: ionizing radiation; NC: negative control; NR: nonresponder; R: responder. On: on‐treatment. Pre: pretreatment.

We used small interfering RNA (siRNA) to knock down the expression of NRG1 in SiHa, a CSCC cell line (**Figure** **6**a). Cell viabilities, measured by Cell Count Kit −8 (CCK‐8) assay, were significantly decreased after NRG1 knock down and these decreased to a greater extent after radiation treatment (Figure 6b). Consistently, colony formation assay indicated that the number of cell colonies reduced after NRG1 downregulation and radiation exposure led to a greater reduction in the number of cell colonies (Figure 6c). These in vitro experiments validated the importance of NRG1 in cell proliferation and radiosensitivity of CSCC cells. To demonstrate solidarity of our observations, we investigated the relevance of NRG1 expression in the response to radiotherapy and prognosis of CSCC patients in cohort 3 (91 tumor specimens from 59 patients, Table S4, Supporting Information). We observed higher protein expression levels of NRG1, determined by immunohistochemistry (IHC), in tumor samples from nonresponders and after radiotherapy treatment (Figure 6d,e). Survival analyses indicated worse overall survival and progression free survival rate in patients with higher NRG1 expression levels (Figure 6f).

Furthermore, we performed multiplex immunofluorescence (mIF) and IHC on pre‐ and on‐radiotherapy treatment FFPE CSCC samples to validate the significance of key gene in IMM program in cohort 3. Similarly, we analyzed expression levels of upregulated IMM genes in cancer cell line encyclopedia data of different fibroblasts cell lines to find potential key genes of IMM program (Figure S11d,e, Supporting Information). But we found that many of the top 20 genes were ribosome gene. Combining this expression data and literature searching, we chose the immediate early response 3 (IER3) gene, which is reported to be expressed in a broad range of human tissues and play complex and divergent roles in cell cycle, differentiation, and survival,^[^ ^31^ ^]^ in our following IHC and mIF experiments.

**Figure 7:**
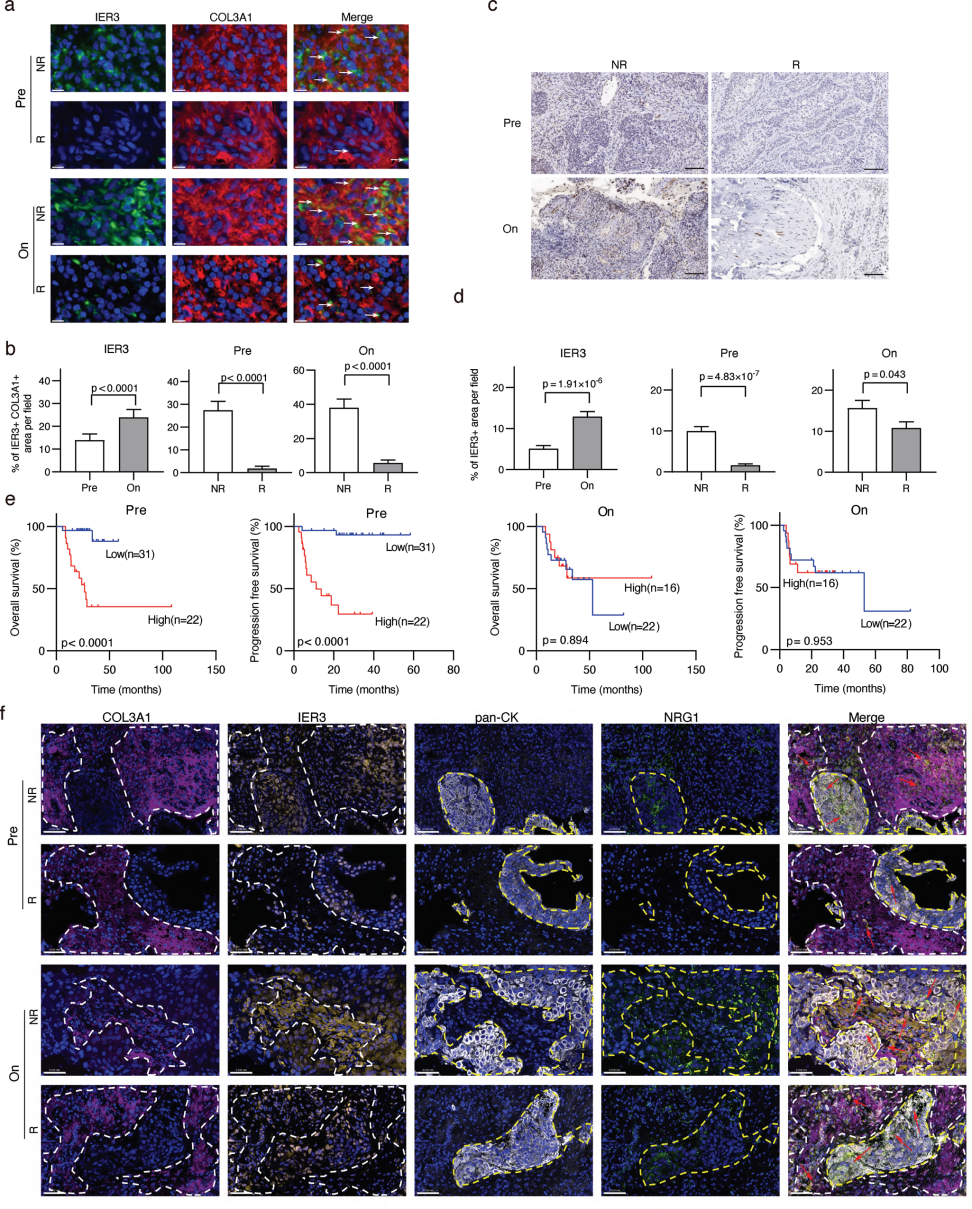
Functional relevance of IER3, an IMM key gene, to radiotherapy efficacy. a) Representative multiplexed immunofluorescence images of CSCC specimens in different groups. Red: COL3A1 (fibroblast marker); green: IER3; blue: DAPI (nuclear stain). Scale bar = 0.05 mm. White arrows indicate cells expressed IER3 and COL3A1. b) Quantification of IER3 and COL3A1 immunofluorescence signals in randomly selected fields by ImageJ software. c) Representative images of CSCC tumor samples through immunohistochemical (IHC) staining with antibody against IER3. Scale bar = 100 µm. d) Quantification of IER3 IHC staining signals in randomly selected fields by ImageJ software. e) Kaplan–Meier curve of overall and progression free survival in CSCC patients stratified by the expression of IER3. f) Spatial analysis of NRG1 in malignant cells and IER3 in CAFs by multiplex immunofluorescence (mIF). DAPI (blue, nuclei), pan‐CK (white, epithelial marker), COL3A1 (purple, fibroblasts marker), NRG1 (green, NRP key gene), IER3 (gold, IMM key gene) were presented in the mIF staining images. Scale bar = 0.05 mm. White circles indicate fibroblasts and yellow circles indicate malignant cells. Comparison between two groups is conducted by two‐sided Wilcoxon rank‐sum test. NR: nonresponder; R: responder. On: on‐treatment. Pre: pretreatment; CAF: cancer associated fibroblasts.

To confirm that IER3 was expressed in CAFs, we used antibodies against IER3 and collagen type III α 1 chain (COL3A1, a marker of fibroblast) to stain a subset of tumor specimens from cohort 3 (19 samples from 11 patients, **Figure** **7**a). Based on mIF staining signals, we found that proportions of IER3+ CAFs were higher in tumor samples from on‐treatment and nonresponder group (Figure 7b). Next, we employed IHC to determine IER3 expression levels in entire cohort 3. Consistent with our mIF results, positivity of IER3 was significantly higher in tumors after radiotherapy treatment and from nonresponders (Figure 7c,d). And we observed that higher IER3 expression levels in pretreatment tumors were associated with worse overall and progression free survival rate (Figure 7e).

Next, we performed mIF staining with antibodies against NRG1 (NRP key gene), IER3 (IMM key gene), pan‐cytokeratin (epithelial cell marker), and COL3A1 (fibroblast marker) on FFPE CSCC samples to investigate the significance of spatial localization of key IMM and NRP genes. We observed that more IER3+ CAFs and NRG1+ malignant cells were concurrently present in CSCC tumors after radiotherapy treatment. In addition, the distances between IER3+ CAFs and malignant cells had the trend to be shorter in both pre‐ and on‐treatment tumor samples from nonresponders after radiotherapy (Figure 7f). In tumor specimens from responders, we observed smaller numbers of IER3+ CAFs and NRG1+ malignant cells in randomly selected fields and a dispersed distribution pattern of IER3+ CAFs inside tumor bed. However, more IER3+ CAFs accumulated near NRG1+ malignant cells in tumor specimens from nonresponders (Figure 7f). Spatial analysis suggested that increased IER3 expression in CAFs and NRG1 in malignant cells, located adjacent to each other, might potentially lead to altered cell‐cell interactions and induce radiotherapy resistance in CSCC. In summary, these results consistently validated that the expression of NRP in CSCC was associated with the efficacy of radiotherapy.

## 3 Discussion

Our analysis of snRNA‐seq and bulk RNA‐seq on matched pre‐ and on‐treatment tumor specimens from two independent locally advanced CSCC cohorts depicted the temporal dynamic landscape of the cell types and states during radiotherapy. These studies also illustrated the transcriptomic remodeling‐associated changes that occur upon exposure to radiotherapy and showed the response of malignant cells and CAFs to the treatment. We consistently identified that high expression of NRP program was related to patients with a worse response to radiotherapy in both independent cohorts. In addition, TCGA data also showed a higher NRP program expression was associated with the worse patient outcomes, and this confirmed the solidity and generality of our conclusions. To our knowledge, the present study is the first to show multicellular dynamics that are associated with the treatment efficacy of CSCC before and during radiotherapy at the single‐cell level.

The potential mechanisms of NRP program expression led to a decrease in sensitivity to radiotherapy and may be the results of the tumor facilitating and drug sensitivity‐related functions of several program genes. For example, Cystic Fibrosis Transmenbrane conductance Regulator (CFTR) regulates cervical cancer cell proliferation and invasion.^[^ ^32^ ^]^ RALY RNA binding protein Like (RALYL), which is RNA‐binding protein, induces HPV16 gene expression.^[^ ^33^ ^]^ The SRY‐related high mobility‐group box 6 (SOX6) could promote cervical cancer cell autophagy and reduce the sensitivity of chemotherapy.^[^ ^34^ ^]^ Secreted phophoprotein 1 (SPP1) is significantly associated with worse prognosis of cancer patients.^[^ ^35^ ^]^ NRP1 has roles in nervous and vascular development, immunity, and tumorigenesis in various cancer types^[^ ^36^ ^]^ and ATP‐binding cassette subfamily B member 1 (ABCB1) has been shown to regulate drug efflux.^[^ ^37^ ^]^ Expression levels of NRP in on‐treatment malignant cells of both responders and nonresponders were increased when compared with pretreatment malignant cells, and malignant cells of the nonresponders expressed the highest level of NRP program (Figure 2f,g). Moreover, in vitro functional experiments showed that knockdown of NRG1, a key gene in NRP program (Figure S11a–c, Supporting Information), facilitated the cell‐killing effects of radiation in CSCC cells (Figure 6). Considering these results, it is more likely that NRP could be a potential driving factor of nonresponsiveness to radiotherapy in cervical cancer patients. Future functional studies in preclinical models should provide further insights into the precise mechanisms involved in our findings.

We did not observe a significant treatment‐associated alteration of the nonmalignant epithelial cells in our snRNA‐seq data (Figure S2d, Supporting Information), but this may have been due to the limited number of nonmalignant epithelial cells recovered from locally advanced tumor samples. In future studies, specimens obtained from early‐stage CSCC patients may provide more insights regarding nonmalignant epithelial cell characteristics. The proportion of the PI16+ iCAF subcluster (Figure 3c), with a higher expression of NRT and IMM programs, was increased in tumors during radiotherapy treatment. In addition, interactions between neuroligin 1 (NLGN1, one of NRT program genes) in CAFs and neurexin 3 (NRXN3, one of NRP program genes) in malignant cells were significantly increased in on‐treatment tumors when compared to pretreatment ones (Figure 4a). However, we only observed consistently significant associations between IMM expression and treatment response and prognosis (Figure 5d–f). These observations may suggest CAFs largely functioned as inflammatory regulators in radiotherapy sensitivity in the cervical cancer setting, which are in line with observations in rectal cancer that inflammatory CAFs are associated with poor responses to radiotherapy.^[^ ^38^ ^]^ However, in‐depth functional studies are needed to further investigate the role of NRT expression in CAFs during radiotherapy treatment.

Although this study expanded our understanding of the radiotherapy response by employing matched pre‐ and on‐treatment samples, we were limited by technical challenges of our snRNA‐seq approach, such as limited ability to capture immune cells. Nevertheless, snRNA‐seq is compatible with frozen specimens and may be more efficient at recovering stromal and epithelial cell types, which are currently less studied. Analysis of immune subsets via other methods, such as multiplex immunofluorescence, may be a potential complementary strategy that may broaden the application of snRNA‐seq. On the other hand, the overall sample sizes of our three cohorts were limited, because we wanted to collect paired pre‐ and on‐treatment tumors. We took advantage of TCGA cervical cancer dataset to cross validate our results from our two independent cohorts. Though we observed consistently increased NRP program expression in malignant cells from nonresponders after radiotherapy alone or radiochemotherapy treatment, the number of patients received radiochemotherapy is limited in snRNA‐seq cohort. More patients treated by different radiotherapy regimen are needed to reach general conclusions. Our future investigations will include larger validation cohorts in an effort to extend these studies.

In summary, our high‐dimensional single‐nucleus molecular profiles shed new light on the radiotherapy‐associated transcriptomic reprogramming and cellular composition that occur in tumors. These novel findings provide knowledge regarding the radiotherapy‐induced cellular responses that occur in patients with CSCC. They can be utilized to maximize the clinical benefits to patients who suffer from locally advanced cervical cancer.

## 4 Experimental Section

### Patient Cohorts

Three independent cohorts for this study were collected. In one cohort, cervical cancer tissues were collected from 11 locally advanced CSCC patients, 8 of which had paired pre‐ or on‐radiotherapy treatment specimens, for single‐nucleus sequencing and bulk RNA‐seq. The second cohort included 17 locally advanced CSCC patients (14 of which had matched pre‐ and on‐radiotherapy treatment samples) for bulk RNA‐seq. The cohort 3 consisted of 59 CSCC patients were collected for IHC analysis, among them 32 patients had paired pre‐ and on‐radiotherapy treatment samples, 21 had pre‐ and the rest 6 patients had on‐samples. All patients agreed and signed informed consent before recruitment to the study and they received no treatment prior to their biopsies. The Ethics Committee of Ren Ji Hospital, Shanghai Jiao Tong University School of Medicine, approved the studies (KY2021‐268‐B). All biopsies were obtained from patients diagnosed with locally advanced CSCC, before treatment (designated as “pre”) or at the 11th fraction of external beam irradiation (designated as “on”). Responders were defined as patients who became completely responsive or near to completely responsive (≥ 90% shrinkage, with negative biopsy) according to the RECIST criteria 3 months after radiotherapy, and they did not progress during the follow up of at least 2 years. Nonresponders were defined as patients whose disease remained either stable or progressed (these had a stable or progressive disease as judged according to the RECIST criteria) or they had a positive biopsy at 3–6 months after radiotherapy and they progressed during the follow‐up. All nonresponders progressed during 2 years (range: 0.3–1.7 years). The details are given in Tables S1, S3, and S4 (Supporting Information).

### Nucleus Extraction and Single‐Nucleus RNA Sequencing

All the banked cervical cancer tissues were quickly frozen in liquid nitrogen for 30 min and then stored in −80 °C refrigerator or in liquid nitrogen. The nuclear fraction was isolated using a Shbio Cell Nuclear Isolation Kit (Shbio, 52009‐10, Shanghai, China)^[^ ^39^ ^]^ and Nonidet P40 with salts by following the Tris nuclear isolation method.^[^ ^40^ ^]^ SnRNA‐seq libraries were prepared using the Chromium Single Cell 3ʹ Reagent Kit v3 (10 × Genomics, USA, 1 000 121) by following the manufacturer's instructions. The Shanghai Biotechnology Corporation then sequenced the libraries obtained.

### SnRNA‐seq Data Preprocessing

The CellRanger 2.1.0 pipeline with the recommended parameters was used to process all the reads and then the FASTQs generated from Illumina sequencing output were aligned to the human genome (version GRCh38) by using the STAR algorithm.^[^ ^41^ ^]^ The output of this pipeline was a gene‐barcode matrix containing the barcoded cells and gene expression counts. Then the CellBender^[^ ^42^ ^]^ “remove‐background” function with default parameters was used to remove ambient RNAs and any technical artifacts. The doublets were identified and removed via DoubletFinder^[^ ^43^ ^]^ v2.0.3. The Seurat (v4.0.5) R toolkit was used for downstream snRNA‐Seq analysis.

### Dimensionality Reduction, Clustering, and Annotation

The profiles from all the specimens into a single Seurat object and after normalizing the data were merged, batch effect correction via Harmony was performed.^[^ ^16c^ ^]^ Next, the uniform manifold approximation and projection (UMAP)^[^ ^44^ ^]^ was used to visualize the individual nuclei profiles and the canonical marker genes expression listed below were used to annotate the cell types. These included fibroblasts (COL1A1 and COL1A2), endothelial (RAMP2, FLT1, and CLDN5), epithelial (EPCAM, KRT5, KRT14, and CDH1), T (CD3G and CD3E), myeloid (FCGR3A, CD14, MARCO, and CD68), B (CD19 and CD79A), and mast cells (GATA2, MS4A2, and KIT). Pathway scores were calculated for each cell using *gsva* function in GSVA software package via ssGSVA function.^[^ ^45^ ^]^ A well‐established gene list (Table S2, Supporting Information) of malignant cell and fibroblast programs from a published study was employed,^[^ ^16a^ ^]^ which utilized similar single nucleus sequencing platform as them. Briefly, they repeated consensus non‐negative matrix factorization 50 times and obtained a set of programs. The optimal number of programs was determined by stability and reconstruction error (Figure S4, Supporting Information). Averaged expression across all genes in the program was determined as program score.

### Copy Number Variation Analysis of Single‐Nucleus Profiles

Epithelial cells into malignant and nonmalignant cells based on the copy‐number alteration as determined by the R inferCNV software package (https://github.com/broadinstitute/inferCNV) were classified.

### Bulk RNA‐seq and Analysis

Bulk RNA‐seq analysis on fresh‐frozen CSCC tissues from 28 patients (50 samples) and 22 patients with matched pre‐ and on‐radiotherapy was employed. The Shanghai Biotechnology Corporation performed the library construction and sequencing. a Qubit 2.0 Fluorometer (Life Technologies, USA) was used to quantify the purified libraries, and an Agilent 2100 bioanalyzer (Agilent Technologies, USA) to validate these libraries. They were then sequenced on the Illumina HiSeq X‐ten (Illumina, USA). The clean data from sequencing FastQ raw data were used by filtering out the rRNA reads and removing the adapters and the short fragment and low‐quality reads in order to perform the downstream analyses. Then the Hisat2 (version: 2.0.4) software was utilized to map the cleaned reads to the human GRCh38 reference genome.^[^ ^46^ ^]^

### Cell–Cell Interaction Analysis

The iTALK R software package^[^ ^16^, ^25^ ^]^ was employed to detect pathway signals between the major cell types in CSCC tumors based on built‐in receptor–ligand pair databases from iTALK. It was considered that the ligand–receptor pairs with *p*‐values < 0.05 were significant differential interactions.

### Cell Type Abundance Estimation of Bulk RNA‐seq

The CIBERSORTx software package was utilized,^[^ ^26^ ^]^ which is a digital cytometry tool and a machine learning method, which allowed to infer cell‐type‐specific gene expression profiles between different data by minimizing the platform‐specific variation, to detect the relative cell types abundance defined by the single‐cell data in the bulk RNA‐seq validation data.^[^ ^26^ ^]^

### Survival Analysis

The gene expression data of CESC patients (306 cases) and their relevant clinical information from TCGA data portal (https://portal.gdc.cancer.gov/) were downloaded. Then the Kaplan–Meier method was used to analyze TTP, DFS, overall survival and progression free survival as the indicated groups.

### Cell Culture, Transfection, CCK‐8, and Colony Formation Assays

The SiHa cell line was bought from the cell bank of the Chinese Academy of Sciences (Shanghai, China). Cells were cultured in MEM (Gibco) supplemented with 10% fetal bovine serum (FBS, Gibco) and 1% penicillin/streptomycin under a 5% CO_2_ atmosphere at 37 °C. The NRG1 siRNA mixture and siNC were purchased from Biotend (Shanghai, China) and transfected to SiHa cells by Lipofectamine 2000 transfection reagent (Invitrogen, ThermoFisher Scientific, Waltham, MA). The efficiency of siRNA was determined by western blot at protein levels and real‐time PCR at mRNA levels as the previous study.^[^ ^47^ ^]^ CCK‐8 (Dojindo, Japan) reagent was utilized to assess cell viability and proliferation and the absorbance values at 450 nm were measured by using the microplate reader. Colony formation detection was performed in a 6‐well plate. The pictures were photographed by a digital scanner and evaluated by ImageJ software (Fiji, 2.12.0).

### Immunohistochemical and Multiplex Immunofluorescence Staining of Formalin‐Fixed, Paraffin‐Embedded Tissue

FFPE tumor tissues in cohort 3 were used for IHC and mIF staining. After the deparaffinization, hydration, and antigen retrieval steps, corresponding primary antibodies (details for antibodies were listed in Methods, Supporting Information) and horseradish peroxidase‐conjugated goat antirabbit secondary antibody (Abcam, ab6712) were added to each section. For IHC, sections were stained by 3, 3'‐diaminobenzidine and then counterstained with hematoxylin and analyzed by ImageJ software (Fiji, 2.12.0). For mIF, 3DHISTECH scanner (Hungary) was used to acquire images and necessary analysis was performed by ImageJ software (Fiji, 2.12.0).

### Statistical Analysis

Statistical analysis was performed by using R 4.0.3 software package. Comparisons between groups as indicated were performed by using the two‐sided Wilcoxon rank‐sum test and the significance of program enrichment among subclusters was determined by the Kruskal–Wallis test. The survival of patients was assessed by using the Kaplan–Meier analysis. Multiple comparisons were Benjamini–Hochberg adjusted by using the p.adjust function of base R. A *p*‐value and/or FDR of 0.05 or less was considered statistically significant.

## Conflict of Interest

The authors declare no conflict of interest.

## Supporting information

Supporting Information

## Data Availability

The data that support the findings of this study are available from Genome Sequence Archive. Restrictions apply to the availability of these data, which were used under license for this study. Data are available from the authors with the permission of Genome Sequence Archive.
