# Supplementary material for: Single‐Nucleus Transcriptome Profiling of Locally Advanced Cervical Squamous Cell Cancer Identifies Neural‐Like Progenitor Program Associated with the Efficacy of Radiotherapy
Source: Adv Sci (Weinh). 2023 Jul 9;10(25):2300348. doi: 10.1002/advs.202300348 (PMC10477877; doi:10.1002/advs.202300348)
Supplement: Supplementary file 1 — Supporting Information [file ADVS-10-2300348-s001.pdf]

ADVANCED SCIENCE
Open Access

Supporting Information

for *Adv. Sci.*, DOI 10.1002/advs.202300348

Single-Nucleus Transcriptome Profiling of Locally Advanced Cervical Squamous Cell Cancer Identifies Neural-Like Progenitor Program Associated with the Efficacy of Radiotherapy

Lei Zhang, Jun Ma, Di Zhou, Junjun Zhou, Bin Hu, Xiumei Ma, Jianming Tang, Yongrui Bai*, Haiyan Chen* and Ying Jing**

Supplementary Methods

Patient cohorts

We collected three independent cohorts in this study. In first cohort, we collected cervical cancer tissues from 11 locally advanced cervical squamous cell cancer (CSCC) patients, 8 of which had paired pre- or on- radiotherapy treatment specimens, for single-nucleus sequencing and bulk RNA-seq. The second cohort consisted of 17 locally advanced CSCC patients (14 of which had matched pre- and on- radiotherapy treatment samples) for bulk RNA-seq. The third cohort consisted of 59 CSCC patients were collected for IHC analysis, among them 32 patients had paired pre- and on- radiotherapy treatment samples, 21 had pre- and the rest 6 patients had on- samples. All patients agreed and signed informed consent before recruitment to the study and received no treatment before biopsy. The Ethics Committee of Ren Ji Hospital, Shanghai Jiao Tong University School of Medicine approved the studies. Clinical information, including age at diagnosis, survival and progression status, International Federation of Gynecology and Obstetrics (FIGO) stage, was extracted from medical records. All biopsies were obtained from patients diagnosed with locally advanced CSCC, before treatment (designated as "pre") or at the 11 th fraction of external beam irradiation (designated as "on"). Radical radiotherapy was given to all these patients, with external beam radiotherapy to the pelvis with or without the para-aortic lymph node area (depending on the status of the positive pelvic and para-aortic lymph nodes), and sequenced by brachytherapy. No chemotherapy was given to the patients who received snRNA seq, due to either old age, medical complications, patient's refusal or other conditions, which were considered contra-indicated for receiving chemotherapy. Responders were defined as patients who became completely responsive or near to completely responsive (≥ 90% shrinkage, with negative biopsy) according to the RECIST criteria 3 months after radiotherapy, and they did not progress during the follow up of at least 2 years. Non-responders were defined as patients whose disease remained stable or progressed (these had a stable or progressive disease according to the RECIST criteria) or they had a positive biopsy at 3-6 months after radiotherapy and their condition progressed during the follow-up. All non-responders progressed during 2 years (range: 0.3-1.7 years). The details are given in Supplementary Tables 1, 3 and 4.

Nucleus extraction and single-nucleus RNA sequencing

All our banked tissues of locally advanced cervical cancer were quickly frozen in liquid nitrogen

and stored in the −80 °C refrigerator or liquid nitrogen. Nuclei isolation was performed using the Shbio Cell Nuclear Isolation Kit (Shbio, 52009-10, Shanghai, China)[1] and Nonidet P40 with salts and the Tris (NST) nucleus isolation method[2] was utilized. The frozen samples were ground to a liquid state by using a tissue homogenizer. To remove impurities, we used a 40 μm cell sieve to pass through the tissue lysate, which then transferred to a new 2mL EP tube, followed by centrifugation at 500 g at 4 ° C for 5 min to obtain a pellet. PB1, PB2 and PB3 solutions were added in turn and the nuclei were observed at the junction of PB2 and PB3. The nuclei were re-suspended in 50 μL of NB solution and counted in a cell counter (Thermo Fisher, Countess II, America). Next, snRNA-seq libraries were prepared by using the Chromium Single Cell 3′ Reagent Kit v3 (10× Genomics, USA, 1000121) by following the manufacturer's instructions. Briefly, each cell was paired with a bead in a GEM (Gel Beads-in-emulsion) and the beads were loaded with a unique molecular identifier (UMI). These had barcodes and poly-adenylated RNA molecules were hybridized to the beads after exposure to a cell lysis buffer. Each cDNA molecule was tagged at the 5'end with a UMI and this cell label indicated the origin of cells during cDNA synthesis. 10× beads were then subject to second-strand cDNA synthesis, adaptor ligation and universal amplification. We prepared sequencing libraries to enrich the 3' ends of the transcripts. These were then linked with the barcode and UMI by using randomly interrupted whole-transcriptome amplification products. Then the sequencing libraries were quantified using a High Sensitivity DNA Chip (Agilent, 5067-4626) on a Bioanalyzer 2100 and by using the Qubit High Sensitivity DNA Assay (Thermo Fisher Scientific, Q32856). All our libraries were sequenced on a NovaSeq6000 (Illumina) using 2×150 chemistry.

SnRNA-seq data pre-processing

We used the CellRanger 2.1.0 pipeline with the recommended parameters to process all the reads. Then the FASTQs that were generated from the Illumina sequencing output were aligned to the human genome (version GRCh38) by using the STAR algorithm[3]. The output of this pipeline was a gene-barcode matrix containing the barcoded cells and gene expression counts. We used the 'remove-background' function in the CellBender[4] software package with the default parameters to remove ambient RNAs and technical artifacts. Briefly, we filtered out the cells with less than 200 and more than 6500 genes as they were likely to represent cellular debris or doublet/multiplet events. Low-quality cells with greater than 10% of transcripts derived from

mitochondrial genes were also excluded. Doublets were identified and removed by using the DoubletFinder[5] v2.0.3 software. The Seurat (v4.0.5) R toolkit was used for the downstream single nucleus-RNA sequencing data analysis of this output.

Dimensionality reduction, clustering and annotation

We merged the profiles from all the specimens into a single Seurat object. We normalized the data using the SCTransform function into the Seurat software package. We regressed out the cell cycle effect and performed a principal-component analysis (PCA) using the top 2000 highly variable genes. Next, we performed a batch effect correction via Harmony[6]. Subsequently, we built a Shared Nearest Neighbor (SNN) graph based on the top 40 batch-corrected components and performed clustering with the resolution set to 1. We used the uniform manifold approximation and projection (UMAP)[7] to visualize the individual nuclear profiles. We used canonical marker gene expression to identify the cell type classifications. These included fibroblasts (COL1A1 and COL1A2), endothelial (RAMP2, FLT1 and CLDN5), epithelial (EPCAM, KRT5, KRT14 and CDH1), T (CD3G and CD3E), myeloid (FCGR3A, CD14, MARCO and CD68), B (CD19 and CD79A) and mast cells (GATA2, MS4A2 and KIT).

Copy number variation analysis of single-nucleus profiles

We classified epithelial cells into malignant and non-malignant cells based on the copy-number alteration. We analyzed the copy number variation by using the R package inferCNV (https://github.com/broadinstitute/inferCNV). All epithelial cells and 300 randomly selected T cells and myeloid cells were used as input. An additional set of randomly selected T cells and myeloid cells were used as reference controls. We obtained CNV signals from each cell and plotted the cells on a dendrogram. Cells that clustered with control T or myeloid cells were labeled as non-malignant, whereas the rest cells were labeled as malignant.

Bulk RNA-seq and analysis

We employed bulk RNA-seq analysis on fresh-frozen CSCC tissues from 28 (50 samples) and 22 patients with matched pre- and on-radiotherapy. The library construction and sequencing were performed at the Shanghai Biotechnology Corporation. We extracted the total RNA from the above mentioned CSCC fresh-frozen specimens using the RNeasy mini kit (Qiagen, Germany)

by following the manufacturer's instructions. We synthesized paired-end libraries by using the TruSeq® RNA Sample Preparation Kit (Illumina, USA) following the guidance of the manufacturer and purified the poly-A containing mRNA molecules by using poly-T oligo-attached magnetic beads. Then we quantified the purified libraries by Qubit® 2.0 Fluorometer (Life Technologies, USA) and this was validated by using an Agilent 2100 bioanalyzer (Agilent Technologies, USA) to confirm the insert size and calculate the molar concentration. Clusters were generated by cBot with the libraries diluted to 10 pM and then these were sequenced on Illumina HiSeq X-ten (Illumina, USA). Clean data were obtained from sequencing the FastQ raw data to filter out rRNA reads and remove the adapters and the short fragments as well as the low-quality reads. We used the clean data with the highest quality to perform the downstream analyses. We used Hisat2 (version: 2.0.4) software to map the cleaned reads to the human GRCh38 reference genome with two mismatches allowed[8]. After genome mapping, the mapped reads of each sample were assembled by featureCounts in a reference-based approach, and then the TPM was calculated with the 'calculateTPM' function in the scuttle R software package.

Cell-cell interaction analysis

We employed the iTALK R software package[9] to detect interactions between the major cell types in CSCC tumors based on the built-in receptor-ligand pair databases from iTALK. The ligand-receptors from iTALK were classified into 4 categories including cytokines, growth factors, immune checkpoints and others. We analyzed the differential cellular interactions of the growth factor, cytokine and NRP genes between the on- and pre- treatment samples in each patient, and between the responders and non-responders. We considered that the ligand-receptor pairs with p-values < 0.05 were significant differential interactions.

Cell type abundance estimation of bulk RNA-seq

We utilized the CIBERSORTx software package, which is a digital cytometry tool and a machine learning method that allowed us to infer cell-type-specific gene expression profiles between the different data obtained by minimizing the platform-specific variations in order to detect the relative cell types abundance defined by our single-cell data in bulk RNA-seq validation data[10]. We first normalized the bulk RNA-seq data by $\log_2$ (TPM + 1) and then loaded these into

CIBERSORTx for analysis. Next, we performed the relative abundance analysis for all the cell clusters from single-nuclear RNA-seq after establishing the signature matrix. We determined the cell-type specific expression of NRP, NRT, BSL and IMM programs by using the 'ImputCellExpression' function.

Cell culture, transfection, CCK-8 assay and colony formation

We bought the CSCC cell line (SiHa) from the cell bank of the Chinese Academy of Sciences (Shanghai, China). Cells were cultured in MEM (Gibco, United States) supplemented with 10% fetal bovine serum (FBS, Gibco, United States) and 1% penicillin/streptomycin under a 5% CO2 atmosphere at 37°C. The NRG1 siRNA mixture (NRG1-si-1: sense (5-3'): GUGCCCAAAUGAGUUUACUdTdT, anti-sense (5-3'): AGUAAACUCAUUUGGGCACdTdT; NRG1-si-2: sense (5-3'): GCCACUCUGUAAUCGUGAUdTdT, anti-sense (5-3'): AUCACGAUUACAGAGUGGCdTdT; NRG1-si-3: sense (5-3'): GGUGAAUCAAUACGUAUCUdTdT, anti-sense (5-3'): AGAUACGUAUUGAUUCACCdTdT) and siNC (sense (5-3'): UUCUCCGAACGUGUCACGUdTdT, anti-sense (5-3'): ACGUGACACGUUCGGAGAAdTdT) were purchased from Biotend (Shanghai, China) and transfected to SIHA cells via Lipofectamine 2000 transfection reagent (Invitrogen, ThermoFisher Scientific, Waltham, Massachusetts, United States). The efficiency of siRNA was determined by Western blot at protein levels and real-time PCR at mRNA levels as our previous study [11], beta-actin and ACTB were used as internal controls respectively and the following NRG1 primer sets were used: 5′-AGAGCCTGTTAAGAAACTCGC-3′ and 5′-GTCCACTTCCAATCTGTTAGCA-3′. Cell Counting Kit- 8 (CCK-8, Dojindo, Japan) was utilized to assess cell viability and proliferation. Briefly, 3×10^3 cells per well were seeded in 96 well microplates. At indicated time points, 10 μL of CCK-8 solution was added to each well and incubated for 1 hours at 37°C. Then, we measured the absorbance values at 450 nm by using the microplate reader. Colony formation detection was performed in a 6-well plate. Cells in different groups were seeded at optimal density. Fresh medium was changed every three days. After 12 days of ionizing radiation (6 Gy), we fixed the cells with 4% Paraformaldehyde and then stained them with crystal violet (0.5% in 25% methanol). The pictures were photographed by a digital scanner and evaluated by ImageJ software (Fiji, 2.12.0).

Immunohistochemical Staining of Formalin-Fixed, Paraffin-Embedded Tissue

Formalin-fixed, paraffin-embedded (FFPE) tumor tissues in cohort 3 were separately cut into 4-μm sections and mounted on glass slides. The slides were baked at 65 °C overnight, followed by deparaffinization and hydration, a 3% H2O2 solution was used to block endogenous peroxidase activities for 20 min. Subsequently all the slides were incubated with 5% normal goat serum for 1 h at room temperature to prevent nonspecific antibody binding, and then incubated at 4 °C overnight with anti-NRG1 and anti-IER3 primary antibody (Abcam, ab191139 and ab65152), 1:200, respectively. Following washes with TBST for three times, Next day, the slides were incubated with HRP-conjugated goat anti-rabbit secondary antibody (Abcam, ab6712) for 1 h at room temperature. Sections were stained by DAB and then counterstained with hematoxylin and we used ImageJ software (Fiji, 2.12.0) to analyze.

Multiplex immunohistochemistry of FFPE tissue

Multiplex immunohistochemistry was performed according to the manufacturers' instructions. Briefly, FFPE tissue sections were subjected to heat at 62° C for 1h. After the deparaffinization and hydration, each section was incubated with H_2O_2 (100μL) at room temperature (25° C) for 10 min and washed the sections with PBS. Antigen retrieval was then performed and 5% BSA were added to the sections for 20 min. We further incubated the sections with 100μL primary antibody (pan-CK (ab234297, 1:2000), COL3A1 (ab7778, 1:1000), NRG1 (ab191139, 1:400) or IER3 (ab65152, 1:400)) from Abcam at 4°C overnight. After washing with PBS, the secondary antibody (Abcam, ab6712) were utilized for 30 min at 37° C and followed by fluorophore-conjugated Tyramide tyramine conversion reagent. The sections were then washed with PBS, repaired with citric acid restorative solution and then treated with the second type of 100μL primary antibody at 4° C overnight. After incubating with the secondary antibody and subsequently fluorophore-conjugated different Tyramide tyramine conversion reagent, the sections were treated with third type of 100μL primary antibody at 4° C overnight as above. The fourth antibody and so on. Finally, 100μL anti-fluorescence quenching blocking reagent (with DAPI) (Beyotime Biotechnology, P0131) was added to each section. Images were acquired by using 3DHISTECH scanner (Hungary) and necessary analysis were performed by ImageJ software (Fiji, 2.12.0).

Supplementary Table 1. The clinical characteristics of patients with locally advanced squamous cell carcinoma analyzed by both single nucleus RNA-Seq and bulk RNA-Seq (cohort 1)

Patient ID	Age (Years)	FIGO 2018 stage	Time of sample collection	Status at last follow up	Response group
Pt1	69	IIb	Pre / on	NED	R
Pt2	62	IIIc1	Pre	DWD	NR
Pt3	57	IVa	Pre / on	AWD	NR
Pt4	51	IIIc1	Pre	NED	R
Pt5	57	IVa	Pre / on	NED	R
Pt6	84	IIb	Pre / on	NED	R
Pt7	65	IVa	Pre	DWD	NR
Pt8	67	IIIb	Pre / on	AWD	NR
Pt9	67	IIIc1	Pre / on	DWD	NR
Pt10	73	IIb	Pre / on	NED	R
Pt11	64	IIIc1	Pre / on	AWD	NR

NED: No evidence of disease; AWD: alive with disease; DWD: dead with disease; R: responder NR: non-responder

Supplementary Table 2. The gene list of programs used for malignant cells and fibroblasts.

Malignant cell lineage programs		Fibroblast programs			
Basaloid	Neural-like progenitor	Adhesive	Immunomodulatory	Myofibroblastic	Neurotropic
IGF2	KCNJ16	NFATC2	SLC22A3	ADAMTS12	SCN7A
CST6	ZBTB16	EMP1	XKR4	CASC15	NFIA
CRYAB	CTNND2	MIR222HG	ANKRD29	POSTN	C7
CST4	PDE3A	SAMD4A	SLCO2B1	NTM	PID1
FBXO2	PDGFD	LMNA	LAMA3	LINC01429	C1orf21
CHPF	CNTN4	GPRC5A	ABCC3	NREP	MAMDC2
LGALS1	CFTR	MMP19	LAMC2	PDGFC	CLMN
ALDOA	FLRT2	MEDAG	GRIN2B	LEF1	PREX2
MT1E	ADCY5	NFATC1	RBM47	NUAK1	MTUS1
ISG15	C6	TSC22D2	NOL4	COL1A1	ADAMTS9-AS2
CCDC85B	CRISP3	LRRFIP1	CP	KIF26B	KCNIP1
LY6K	RALYL	RFX2	KEL	NOX4	LAMA2
KRTAP2-3	NR1H4	PFKP	ZNF804B	FN1	EBF1
MT2A	BCL2	PTPRJ	TNC	SULF1	ABCA6
CKAP4	ESRRG	ANKRD28	ACTB	COL1A2	NID1
PRNP	SLC4A4	CAV1	TMEM108	WNT5A	EPHA3
IFI27	CSMD2	TEX26-AS1	TMEM178B	COL3A1	IL1RAPL1
DKK3	RGS17	CDH2	CCL21	COL11A1	TMEM132C
C9orf16	CRP	ANXA2	ABCB11	CDH11	SPTBN1
GJA1	SLC17A4	CTNNAL1	SLCO2A1	NKD1	ADAMTSL3
IFI6	RELN	SLC19A2	IL15	DOCK4	NEGR1
CRIP1	PAH	CRY1	FDCSP	PLPP4	AC016831.7
POLR2L	PKHD1	CNN1	MUSK	MMP11	SLC9A9
THBS1	LINC01320	SYN3	PLA2G4C	ADAMTS14	MIR99AHG
LGALS7	ACSM3	ANXA5	ATP8A1	ADAMTS6	ZBTB20
TNNC2	DSCAML1	TES	ADGRL3	FAP	SRPX
PTMS	AR	LHFPL2	LIFR	RUNX2	ABCA8
ROMO1	ZNF208	LMCD1	NPY1R	RUNX1	TGFBR3
IFI27L2	TTLL7	ERRFI1	ARHGAP15	MGAT5	ABCA10
CD81	SOX6	UGP2	CTSS	SNTB1	PTEN
TUBB	SPP1	LMCD1-AS1	RASGEF1B	KIAA1549L	ZBTB16
LY6E	ASXL3	IQCJ-SCHIP1	BIRC3	CTHRC1	RHOBTB3
C12orf57	POU6F2	ACSL4	NRG2	LINC00578	SLIT2
PSAP	DZIP1	ZSWIM6	JUN	RNF144A	PDK4
PDLIM4	ITIH5	DDAH1	LPAR1	ENC1	FREM1
C19orf53	PRKG1	PTPN1	EXOC3L4	SYTL2	SOX6

CTSZ	IL1R1	ABL2	PTPRF	ITGA1	CACNA1D
SNRPD2	SEMA3E	ESYT2	CR2	DCBLD1	ABI3BP
GPS2	GUCY1A2	GFPT2	CHL1	COL10A1	HMGCLL1
OST4	AC092535.3	ATP13A3	EGR1	CALD1	AOX1
PRDX1	RCAN2	BAIAP2	ANO9	CARMN	MAPK10
NPC2	SLC3A1	GLIS3	SLCO1A2	CHST11	SSH2
VIM	MAN1A1	ERCC1	ZFP36L2	PDZD2	KAZN
TRMT112	WDR72	CD44	OSMR	ANTXR1	ARHGAP10
MZT2B	ACSS3	ENAH	EDNRB	GREM1	AFF3
SCAND1	ONECUT1	SERPINE1	PTMA	INHBA	ARHGAP6
MMP2	NRP1	CLIC4	PLD5	NPR3	ABLIM1
CD59	AKAP7	ATP10A	EHBP1L1	GRIP1	PTPRG
MT1A	LDLRAD4	FNIP2	TIMM23B	SLC6A6	ADGRD1
CFL1	AC012593.1	MYOF	TRAF1	FBXO32	SPARCL1
SEC61G	CALN1	NEDD9	TAGLN	FGD6	FKBP5
TMSB10	UGT2B15	FOSL1	RASGEF1A	SALL4	ABCA9
BANF1	AGBL4	RTN4	EEF1A1	KCND2	ANKS1B
SOSTDC1	GLIS3	COBL	CACNA2D3	ITGA11	COL21A1
VAT1	TRPV6	MYH10	ADRA1A	MIR181A1HG	FRMD3
MRPS12	ABCB1	FOSB	S1PR3	ACTA2	IMMP2L
PPDPF	PLXDC2	KDM6B	IER3	LAMA4	CELF2
NPB	NLGN4Y	CAPN2	PLCXD3	APBB2	ADD3
TIMM8B	NEK10	ANXA1	SLC26A7	EDNRA	CCNH
UBA52	TACC1	YWHAZ	IRF8	FUT8	HAND2-AS1
NBL1	HOMER2	RGCC	NFAM1	BICD1	DSCAML1
CENPB	SNAP25	EGFR	PDE4B	MBOAT2	TFPI
DGCR6	SCTR	HIF1A	SORL1	PALM2-AKAP2	NR2F2-AS1
GADD45GIP1	SLC2A2	SH3RF1	ACHE	SUGCT	BOC
COX5B	MIR99AHG	ELL2	SLC26A3	HIP1	ADGRB3
DYNLL1	TRABD2B	KLF6	TPM4	VSNL1	PDE1A
ANAPC11	NR5A2	WEE1	CDH1	ENTPD1	MKLN1
GPX3	FAM135B	S100A10	CTSH	SGIP1	NFIB
LINC01615	FGG	P4HA3	PAPLN	EEPD1	PBX1
PPIA	DCDC2	HOMER1	SDK1	KANK4	FBLN5
CCND1	SYNE1	TRIB1	DAPK2	FRMD5	CPED1
RABAC1	CRISP2	ADAM12	ACTG1	PPFIBP1	HIF3A
COX6B1	SEMA5A	ITGA5	DEPTOR	FOXP1	PIK3R1
CTGF	BICC1	SLC7A1	CYSLTR2	ADAM19	TENM2
RPS17	TNS1	KCNMA1	DTNA	SIPA1L1	COL4A4
TNFRSF12A	CHST9	TUBB6	COL27A1	FARP1	SESN3
SH3BGRL3	NCAM1	HRH1	CXCL12	PTK7	ITPR1

RPL35	RORA	GEM	TNFAIP2	NHSL1	DLG2
PRDX5	ADAMTS9-AS2	GPR176	NR4A1	VCAN	FBLN1
HSBP1	APCS	PCGF5	LINC01197	HMGA2	SSBP2
TUBA1B	MUM1L1	MICAL2	CR1	EPSTI1	GPHN
TMSB4X	SETBP1	PER2	CSF2RB	CDK6	ADAMTS3
NUPR1	NRCAM	ST6GALNAC5	VCAM1	SPATS2L	SAMHD1
RHOC	CFAP221	DOK5	TMSB4X	PALLD	KCTD3
TMED9	ATP13A4	LOX	LMF1	STAMBPL1	LINC01088
RARRES3	LIN7A	COL12A1	OCA2	RASGRF2	NEURL1B
GAPDH	TTC28	TIMP3	RPS9	MYH9	RUNX1T1
S100A2	STXBP6	ACTN4	TNFRSF1B	ARHGAP31	GPC5
FJX1	KCTD16	FLNB	THBS1	CDKL5	SOX5
MZT2A	NR2F2-AS1	CRIM1	LDLR	TPM1	ADGRL2
UQCRQ	DOCK8	PMEPA1	PTPRT	ATXN1	AFF1
NFE2L1	LRRK2	EFHD2	MYO16	PTPRE	NOVA1
C19orf33	RERG	S100A6	EBF3	ZEB1	PARD3
ATP6V1G1	AC018742.1	PXDC1	TLR1	FAM168A	GFRA1
RPL8	PCDH9	MYO1B	C1RL-AS1	ST6GAL2	CCND3
SDC1	PTCHD4	TAOK3	FOS	COL5A1	FAM13A
HLA-DQB1	ATP10A	MLF1	SERPINB9	FNDC1	MFSD6
SFN	WNK2	UAP1	COL23A1	PLXNC1	RGL1
FAU	DPYD	CORO1C	GNA14	EIF4G3	SETBP1
CALR	AC019117.1	TIPARP	PKP2	ANO1	GHR
TMED2	SNCAIP	ITGB1	FTH1	LRIG3	DYNC1I1
FTH1	LIMCH1	DENND5A	SAT1	TCF4	CCDC102B
NDUFB10	ANXA4	MEF2A	NCEH1	HOXB3	SPATA6
MLLT11	BMPR1B	RNF149	TNFAIP3	APBA2	NSF
EFCAB3	KCNMA1	MKL1	JUND	GULP1	DCN
RPL38	PTP4A1	MYO9B	TPT1	HECW1	LDLRAD3
TUBA1A	DTNA	EXT1	KIAA1671	TLN2	DCLK1
B3GALT6	NBEA	GLUD1	PNISR	SPIN1	RNF13
FSTL1	TTN	FNDC3B	PCOLCE2	IRS1	RORA
GSTP1	DLG2	GADD45B	FGF7	SPON2	NLGN1
RNF181	PTPRM	NUP153	ITIH5	NXN	CECR2
RPL28	SCN9A	HMGA1	UBC	TSC22D1	FOXO3
CHCHD2	TMEM132C	FGFR1	HDAC9	TENM4	COL4A1
MRPL51	HIF1A	FHL2	CYR61	GRIK2	UTRN
RPS13	KHDRBS2	ARC	ADAMTSL1	NPAS2	PIAS1
TMBIM6	SLC16A7	CBLB	GRIA4	STX7	COL4A3
MYL6	AJAP1	MBNL2	GARNL3	BCAT1	CACNB2
UQCR10	KIF12	ACTN1	IL4R	PRICKLE1	COL4A2

RPS19BP1	SEMA6A	FAM155A	SPNS2	ZNF521	FIGN
FTL	GRM8	FSIP1	NBEAL2	ZNF532	FMNL2
NDUFS5	LRAT	GATAD2A	ZFP36	KLHL2	SOBP
POLR2K	TRIM5	CREB5	PNRC1	ITGB5	EGFR
IL32	NFIB	SIK3	PARP14	TNFRSF19	TGFBR2
SAA1	PDE7A	KALRN	TXNIP	ARMC9	FAM102B
RCN1	ONECUT2	CDK17	STRIP2	TNS3	DPYD
COPRS	PRICKLE2	ITPKC	SVEP1	GFPT1	FAM135A
RBP1	CDH6	PDLIM5	FADS1	MRVI1	PPP1CB
KRT17	AC124312.1	PSME4	PLXNA4	WNK1	IRAK3
TXN	MAPK10	NCS1	SLC2A3	MANBA	TMEM144
SLC39A4	RBPMS	MBNL1	LINGO1	TBL1XR1	MGST1
CCDC167	APCDD1	CAMK1D	C7	MIR4435-2HG	LAMB1
PERP	CES1	SGK1	PRRC2C	DNAJC15	ADCY3
TIMP3	LINC01266	RAB11A	DAAM1	SCN8A	PODN
AURKAIP1	SERPINA6	PLAT	CLSTN3	TWIST1	ADH1B
MAF1	ADGRL2	RPS6KA3	CCL19	COG6	PLSCR4
RPS27A	LINC00671	FLNC	INO80D	PCED1B	ABTB2
FBN1	TENM3	YWHAG	ATP8B4	C9orf3	ALDH1A1
P4HB	KCNJ15	ITGAV	ALPK1	MAP3K4	PAK3
ZNF593	MEIS2	MAP2K3	NOVA1	SMC6	NBEA
RPL27	FIGN	IL1R1	COL4A4	DIO2	ITM2B
RPLP2	GABRB3	ADAM17	PITPNC1	TTC3	TNRC6B
PDZRN3-AS1	SDK1	HIF1A-AS2	PCDH11X	ZMYM4	TRERF1
C4orf48	KCNT2	PITPNM2	RPS11	SAMD3	STXBP4
IGFBP6	ZNF503-AS1	TNFRSF12A	APBB1IP	STARD4-AS1	PRKCH
SOD1	CFH	HEG1	TNFSF10	WWC1	SLC8A1
RPS2	ARHGAP44	IQGAP1	RPS6KL1	UNC5B	PITPNC1
ZNHIT2	ANKS1B	VGLL3	PDE1C	LINC01060	CALCRL
LGALS3BP	THSD4	TP53BP2	GAPDH	DGKI	C1S
SLC7A5	NR3C2	PLEKHA7	NFKBIA	BBX	MT1X
B4GALNT1	ADARB2	AXL	CD82	SSH1	JADE1
SERF2	NRXN3	PHF20	TLE2	KCNQ1OT1	PTPRK
CHCHD10	TOX	KIF1B	SRRM2	GXYLT2	PDE7B
HSPA5	NFIA	LPP	TMEM176B	GPR63	ACACB
EXOSC4	ANPEP	MCL1	C3	F13A1	OGFRL1
CHCHD5	GRB10	PPP1R12B	PYHIN1	WLS	CLIP4
CDA	TUSC3	ASAP2	HMCN2	PLS3	GAB1
RPS19	HABP2	GLS	RPS8	SLC24A2	PLEKHA5
H2AFJ	DACH1	ATP1A1	RPL13	EPC1	SMIM14
ATP6V1F	MPP6	CLIP1	RPL10	KIFAP3	TXNIP

TRIM54	CAMK1D	TRIO	IRF3	COL7A1	NCOA1
RPL36	SLC1A1	LINC00968	SMAP2	VGLL4	RBMS1
LDHB	SERPING1	ATP2B4	ADGRE5	PDZRN3	SDCCAG8
TOMM7	MEF2C	PTPN14	PER1	PRDM1	STK24
RPS25	MUC5B	RYBP	PLEC	COL8A1	FOXP2
PEBP1	FHIT	HECTD2	ZDHHC14	ISM1	ABCA9-AS1
RPL41	SLC5A1	ADAM9	PHPT1	ZNF609	COLGALT2
CRABP2	PDGFC	RAI14	EPHB1	CLCN3	KDM6A
FSTL3	ASRGL1	UCHL3	FAM189A1	ADAM22	PTPN13
POPDC3	SULT1C4	DDX21	RPLP0	ACVR1	MATN2
YWHAB	CACNA1H	XYLT1	TXNRD1	TMEM45A	CD47
S100A11	NREP	GREB1L	LINC00092	PRDM6	ARHGAP26
COA3	DSEL	TGFBR1	SPATA6L	ETV6	MBP
VAMP8	SLC4A7	KPNA4	EEF2	AEBP1	DANT2
COX6C	REG1A	DMD	ABHD17C	TANC2	KIF5C
TACSTD2	MLLT3	PDZRN4	ART4	SRPK2	CNKSR2
C1orf122	TDRP	IPO7	NFKB2	DDR2	KCNT2
HLA-C	DLGAP1	SEPT9	ARRDC3	WIPF1	ABCC9
KRT8	ST8SIA3	YBX3	HNMT	PRKD1	RALGAPA1
PODNL1	FXYD2	RAMP1	CLU	SPATS2	IL6ST
EIF1	EPB41L4A	IGF2BP2	SPIC	RFX8	ECHDC2
RPL35A	IQCA1	LDHA	TOM1L1	MSC-AS1	SYNPO2
NDUFB9	PRKCE	ATP11A	FTL	MMP14	TBC1D5
UQCRB	MPP7	PCDH7	MYL6	ZNF292	ELMO1
AP2S1	NRG1	PRKCA-AS1	RPS2	IGFBP5	GFOD1
CYSRT1	ITPR2	SERTAD2	DOCK8	ANO4	MARCH2
RPL19	ZNF667	DENND4A	COLQ	MAML3	FLRT2
NXT1	AUTS2	WISP1	GAK	RAI14	KLF12
SNCG	LRP1B	IL6R	KLHL25	HECW2	RBM26
MARCKS	BCO2	HIPK2	SORCS1	PRR5L	KCNN3
IFITM3	PBX1	HIVEP2	PLXNB2	MDFIC	PDE3A
FAM210B	RASSF8	ACLY	SLC9A9	PCCA	NAALADL2
ISOC2	HYDIN	PLAUR	AHNAK	ETV1	INSR
UQCRHL	PRKD1	ANO6	DDX24	SIPA1L3	AUH
BAD	ZSCAN18	CTNNA1	IL34	CAMK4	PLCL2
FAM83H	KLKB1	PDLIM3	EPAS1	VEZT	CDON
PGAM1	ZNF676	CAV2	FLNB	UHRF2	PTPN12
CREB3	PBX3	MSRB3	SLIT3	GUCY1A2	PRKAG2
BOLA3	CEP112	MTHFD1L	EPHA2	PHF21A	ADAMTS15
ADAD2	CYS1	COBLL1	ETS2	GPC6	OAF
NNMT	GALNT18	DPYSL3	FAM20A	NBAT1	KLHL13

Supplementary Table 3. The clinical characteristics of patients with locally advanced squamous cell carcinoma analyzed by bulk RNA-Seq (cohort 2)

Patient ID	Age (Years)	FIGO 2018 stage	Time of sample collection	Status at last follow up	Response group
Tpt-3	61	IIIc2	Pre / on	DWD	NR
Tpt-14	50	IVa	Pre	DWD	NR
Tpt-15	52	IIIc2	Pre /on	AWD	NR
Tpt-16	54	IIb	Pre	DWD	NR
Tpt-17	73	IVa	Pre /on	AWD	NR
Tpt-18	47	IIIc1	Pre /on	DWD	NR
Tpt-19	47	IIIc2	Pre /on	NED	R
Tpt-20	54	IIIc1	Pre /on	NED	R
Tpt-21	61	IIIc1	Pre /on	NED	R
Tpt-22	46	IIIc1	Pre /on	NED	R
Tpt-23	53	IIIc1	Pre /on	NED	R
Tpt-24	65	IIIc2	Pre /on	NED	R
Tpt-25	69	IIb	Pre /on	NED	R
Tpt-26	68	IIIc2	Pre	NED	R
Tpt-27	75	IIIc1	Pre /on	NED	R
Tpt-28	27	IIIc2	Pre /on	NED	R
Tpt-29	56	IIIc1	Pre /on	NED	R

NED: No evidence of disease; AWD: alive with disease; DWD: dead with disease; R: responder; NR: non-responder

Supplementary Table 4. The clinical characteristics of patients with locally advanced squamous cell carcinoma analyzed by IHC (cohort 3, number of patients=59)

Characteristic		Number
Age (years; median, range)		56, 32-82
FIGO stage	IIb	5
	IIIa	2
	IIIc	37
	IVa	10
	IVb	5
Treatment	RT	12
	CCRT*	47
Tumor control	Recurrence	34
	No recurrence	25
Status at last follow up	Dead	18
	Alive	41

CCRT: concurrent chemoradiotherapy

Supplementary Fig. 1

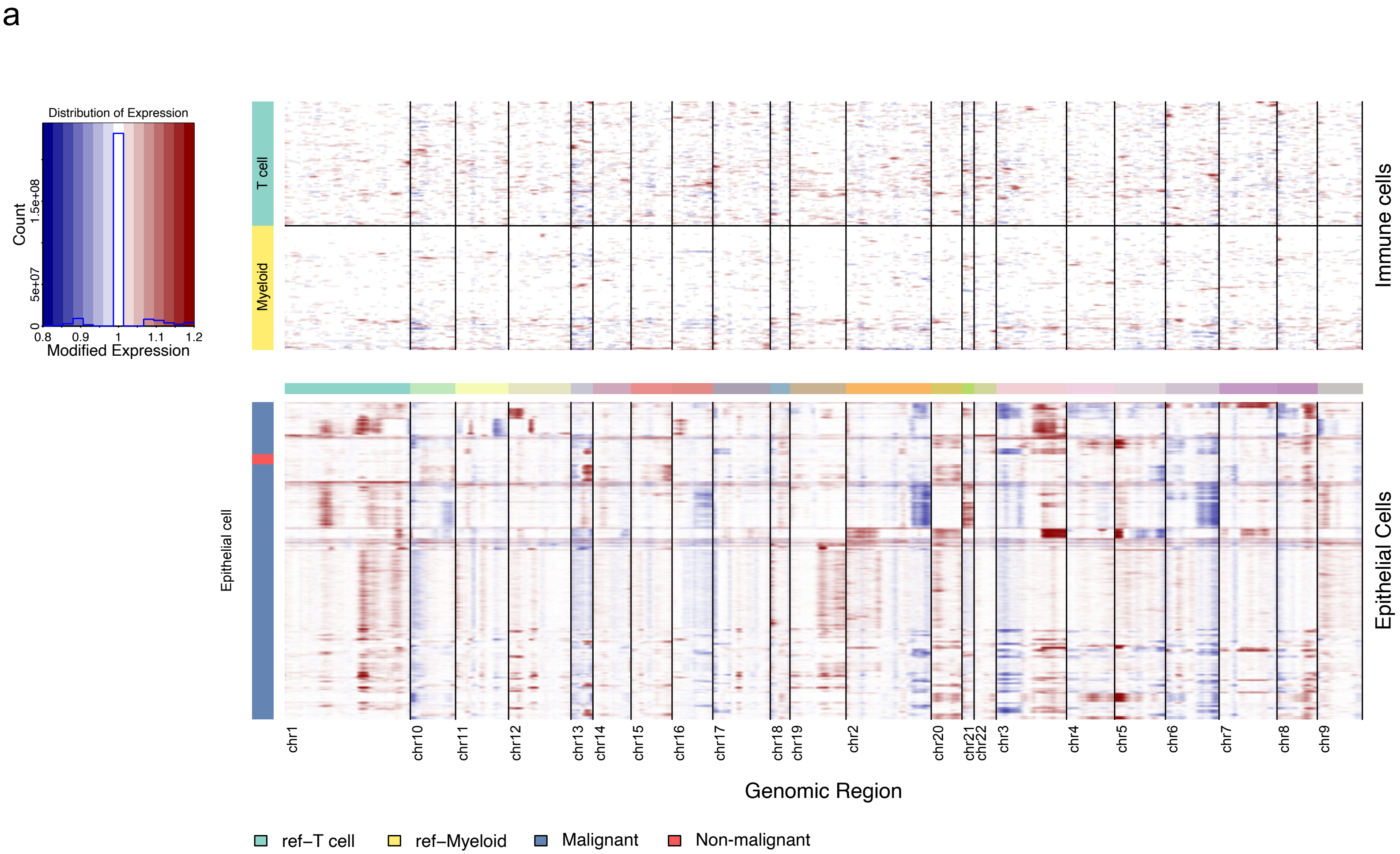

Supplementary Fig. 1. The landscape of inferred large-scale CNVs for all of the epithelial cells.

Supplementary Fig. 2

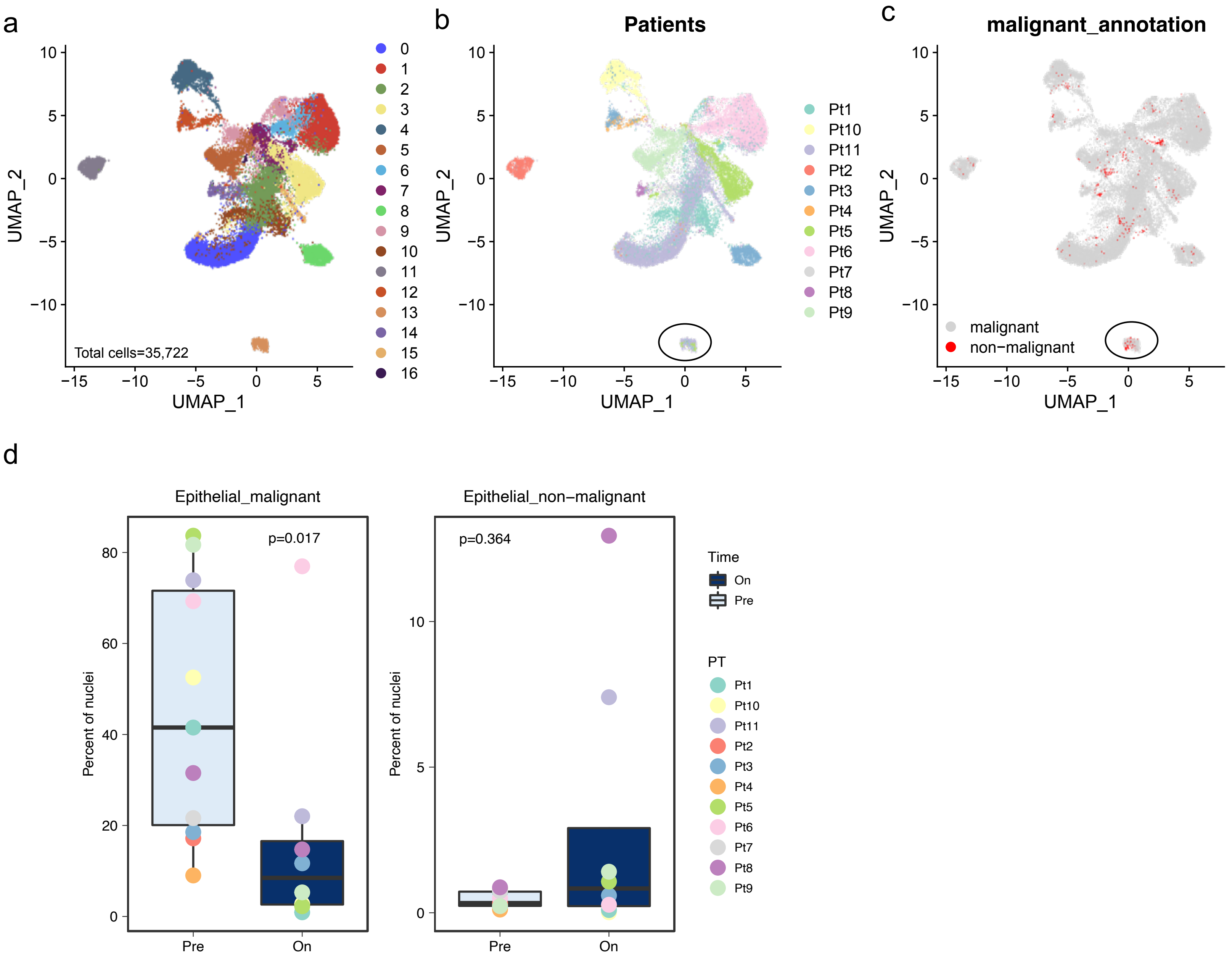

Supplementary Fig. 2. The comparison of malignant and non-malignant epithelial cells. a-c, UMAP projection of epithelial cells isolated from all tumors. Cells are colored by clusters (a), by patients (b) and by malignant annotations (c). d, Comparison of cell type proportion in the different groups. Boxplots indicate the median ± 1 quartile, with whiskers extending from the hinge to the smallest and largest values within 1.5 interquartile range from the box boundaries. On: on-treatment. Pre: pre-treatment. Comparisons were performed by using the two-sided Wilcoxon rank-sum test.

Supplementary Fig. 3

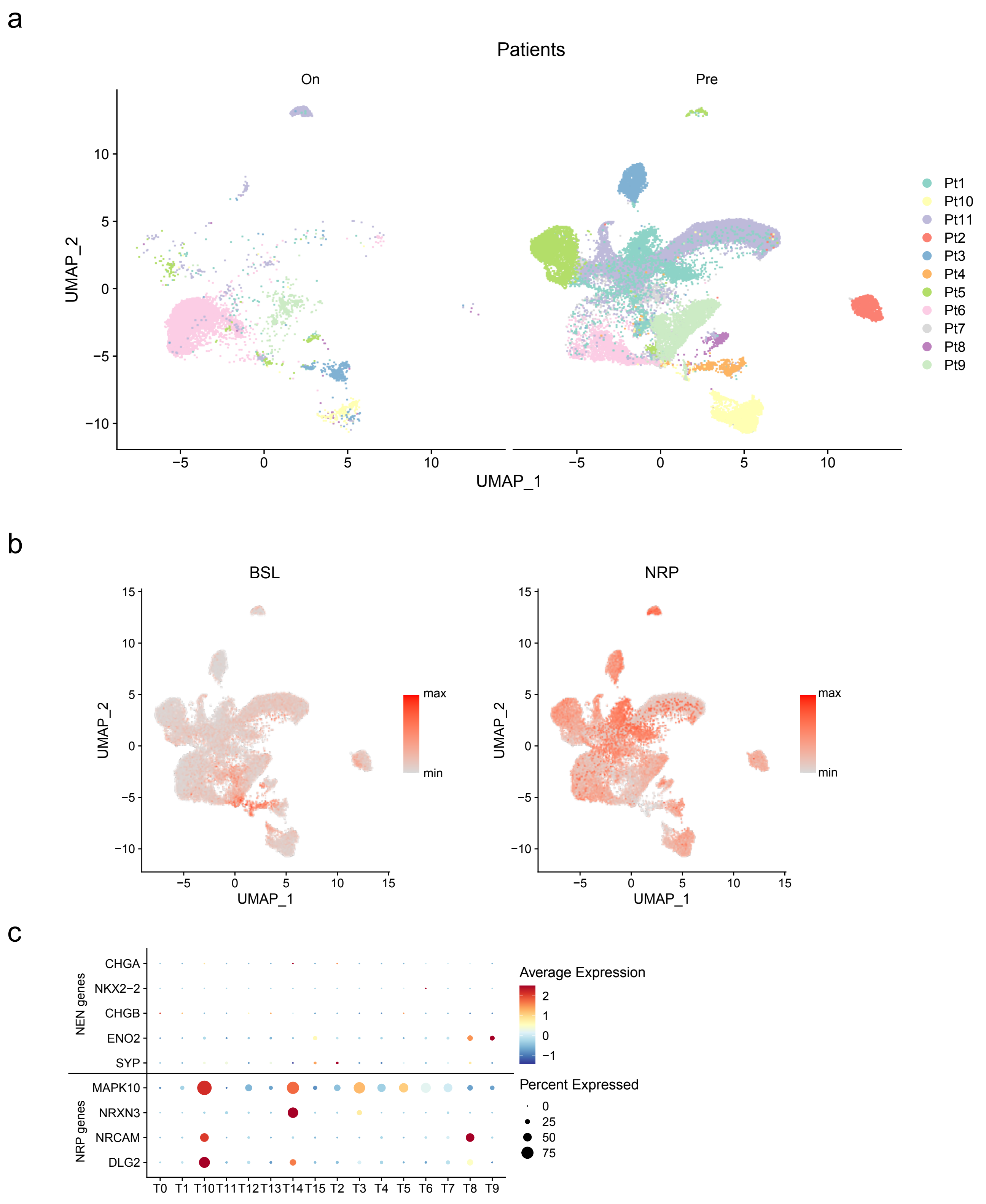

Supplementary Fig. 3. The NRP program expression in malignant cells. a, UMAP projection of epithelial cells isolated from all the tumors. Cells are colored according to patients and are split by treatment status. b, UMAP as in a, showing expression of BSL and NRP programs. c, The expression of specific genes of either NRP or NEN programs in different malignant cell sub-clusters. BSL: basaloid. NRP: neural-like progenitor. NEN: neuroendocrine-like.

Supplementary Fig. 4

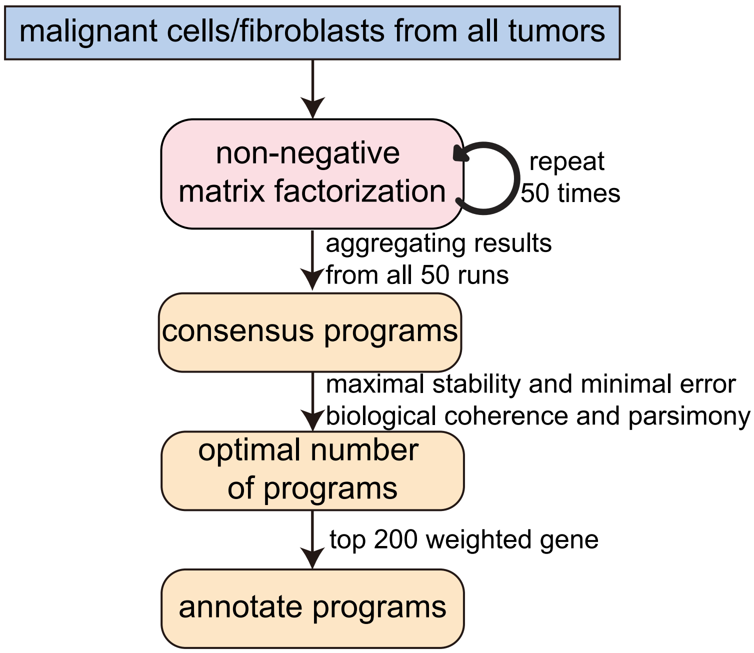

Supplementary Fig. 4. Illustration for lineage program generation.

Supplementary Fig. 5

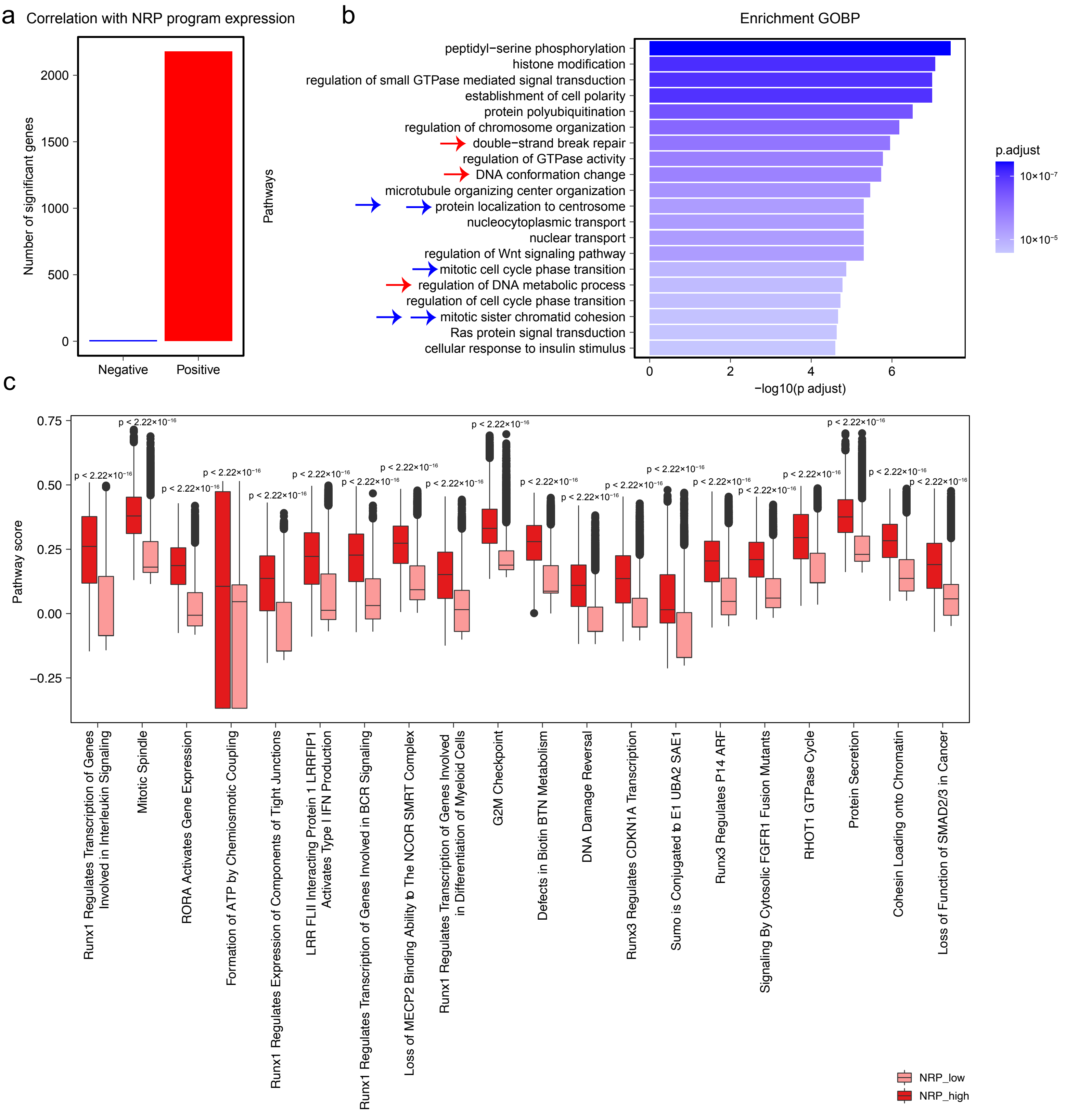

Supplementary Fig. 5. Potential mechanisms associated with NRP program increment in malignant cells. (a) Results of spearman correlation analysis between expression of NRP program and all genes. Significant genes were defined as FDR < 0.05 and absolute Rs value ≥ 0.3. (b) Pathway enrichment results of the genes significantly correlated with NRP program expression. Top 20 pathways were plotted. Red arrows indicate DNA repair related pathways; blue arrows indicate cellular mitosis related pathways. GOBP: gene ontology biological process. (c) Comparison of pathway expression among NRP highly expressed (NRP-high) or lowly expressed (NRP-low) malignant cells. Median value of NRP expression levels in all malignant cells was employed as the cutoff to determine NRP-high and NRP-low cells. NRP: neural-like progenitor; GSVA: gene set variation analysis. *P*-value was calculated by using the two-sided Wilcoxon rank-sum test. Boxplots indicate the median ± 1 quartile, with whiskers extending from the hinge to the smallest and largest values within 1.5 interquartile range from the box boundaries.

Supplementary Fig. 6

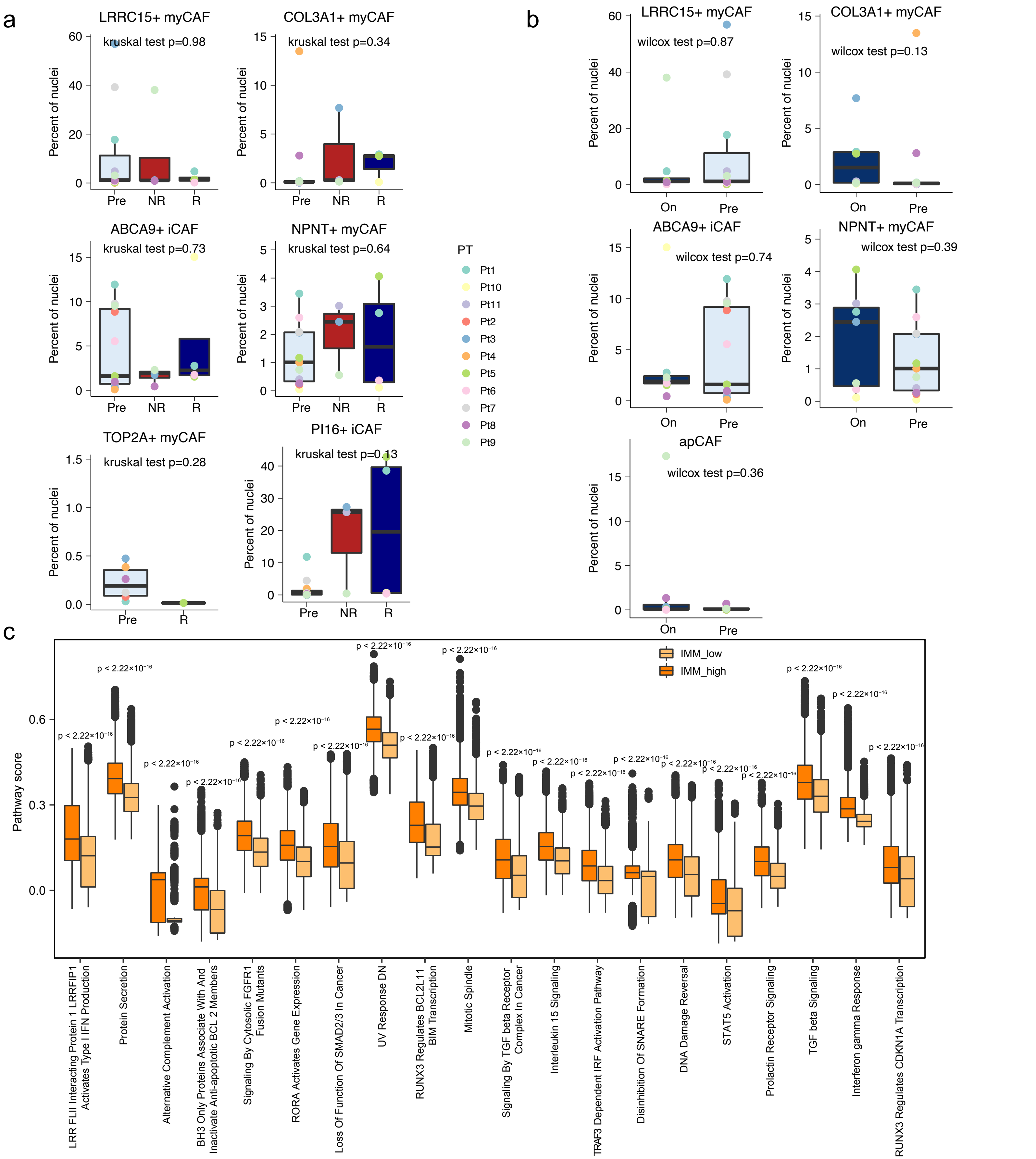

Supplementary Fig. 6. Alteraions in CAF. A comparison of CAF sub-cluster proportions in different groups (a-b). Comparison of pathway expression among IMM highly expressed or lowly expressed CAFs (c). Median value of IMM expression in CAFs was employed as cutoff to determine IMM-high and IMM-low cells. Boxplots indicate the median ± 1 quartile, with whiskers extending from the hinge to the smallest and largest values within 1.5 interquartile range from the box boundaries. On: on-treatment. Pre: pre-treatment. NR: non-responder. R: responder. Comparisons were performed by using the two-sided Wilcoxon rank-sum test or Kruskal-Wallis test.

Supplementary Fig. 7

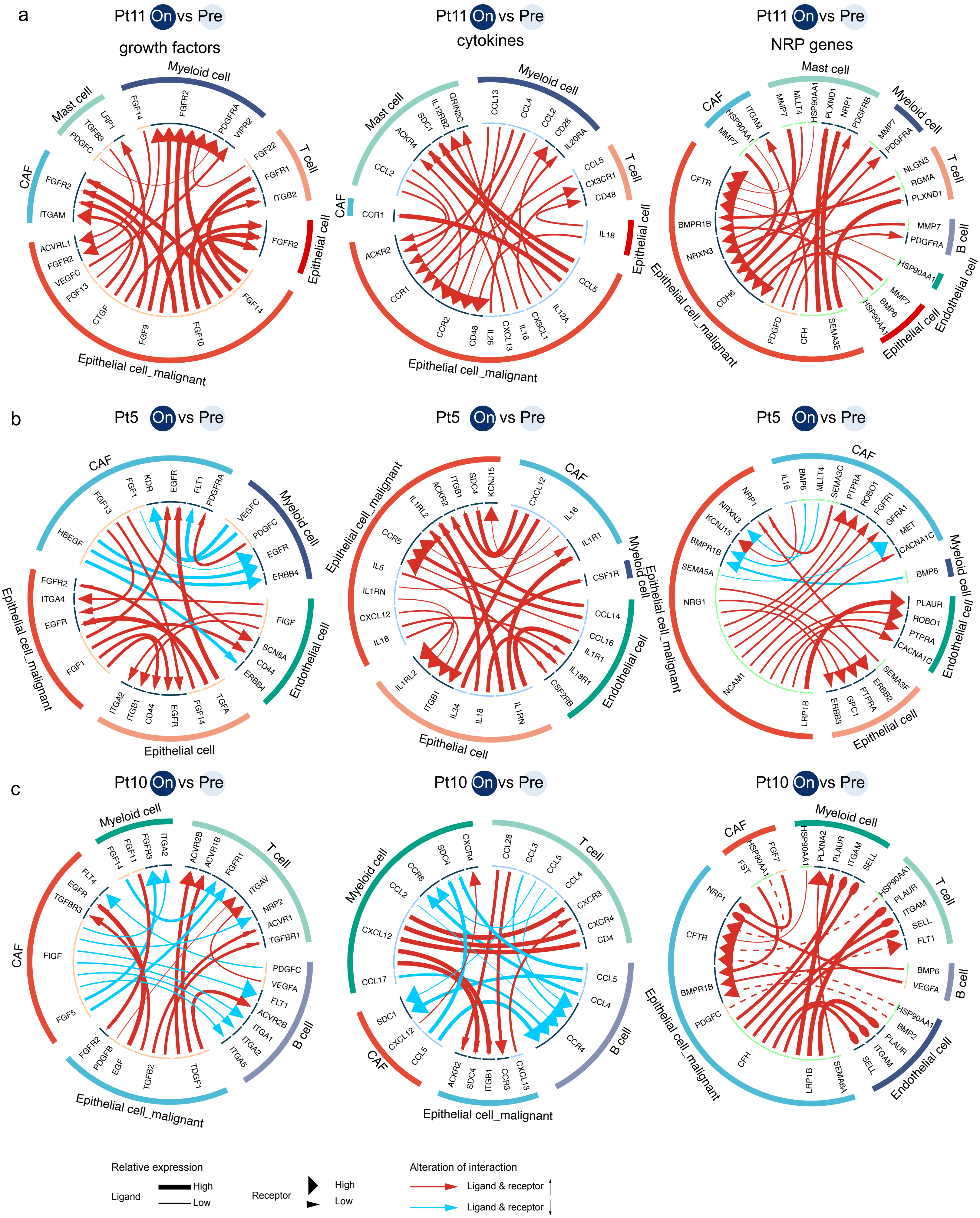

Supplementary Fig. 7. Enriched ligand-receptor cell-cell interactions across the major cell types in radiotherapy-exposed tumors. a-c, Representative circos plots show details of the top 20 differentially regulated growth factor (left), cytokine (middle) and NRP genes (right) ligand-receptor pairs when comparing pre versus on in Pt11 (a) Pt5 (b) and Pt10 (c). The lines are colored by the alterations observed. The length of the lines indicates the relative expression of ligand. The size of the arrows indicates the relative expression of receptor.

Supplementary Fig. 8

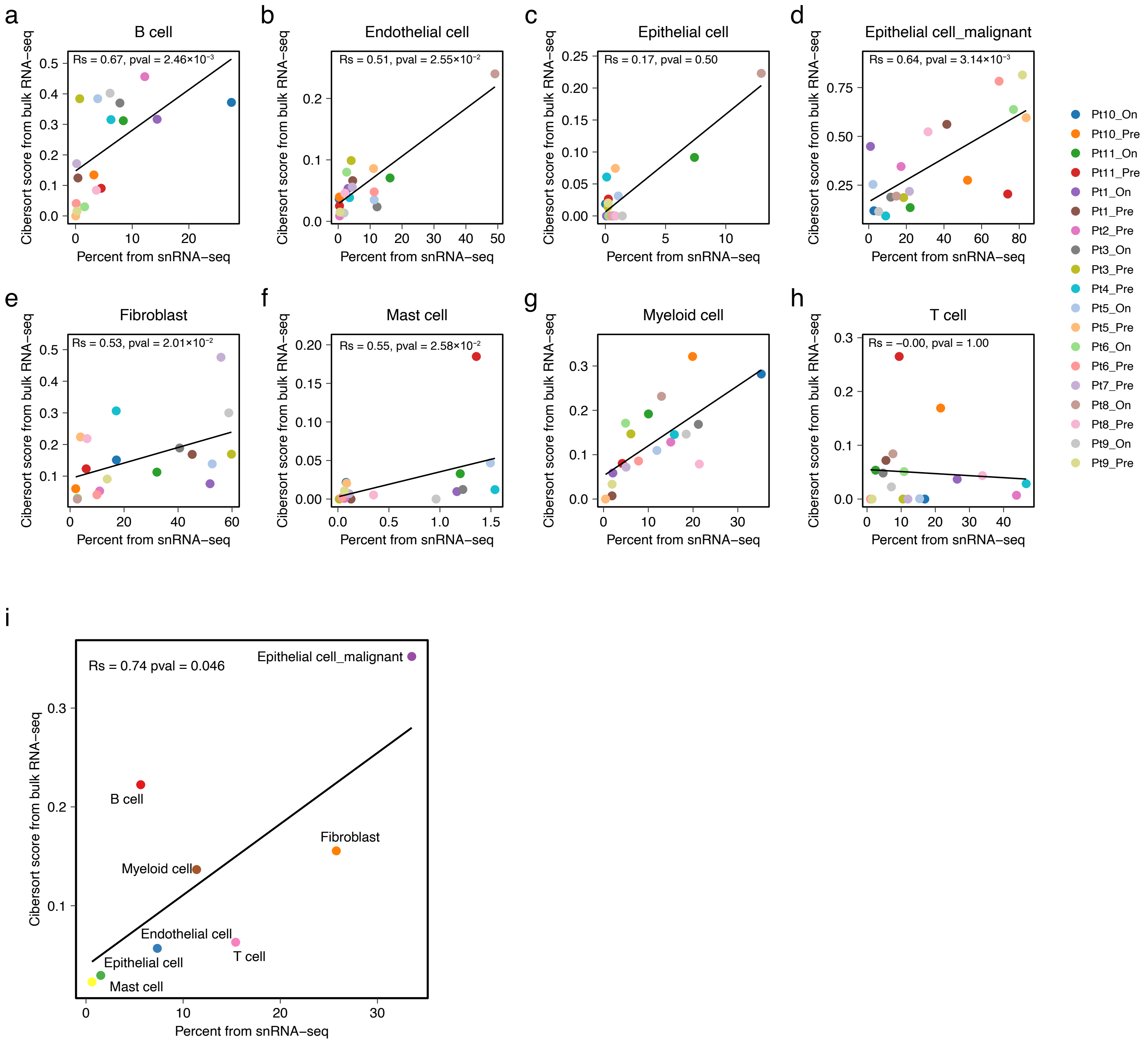

Supplementary Fig. 8. Spearman correlation between CIBERSORTx inferred cell abundance based on the bulk RNA-seq data and the cell percentage based on snRNA-seq.

Supplementary Fig. 9

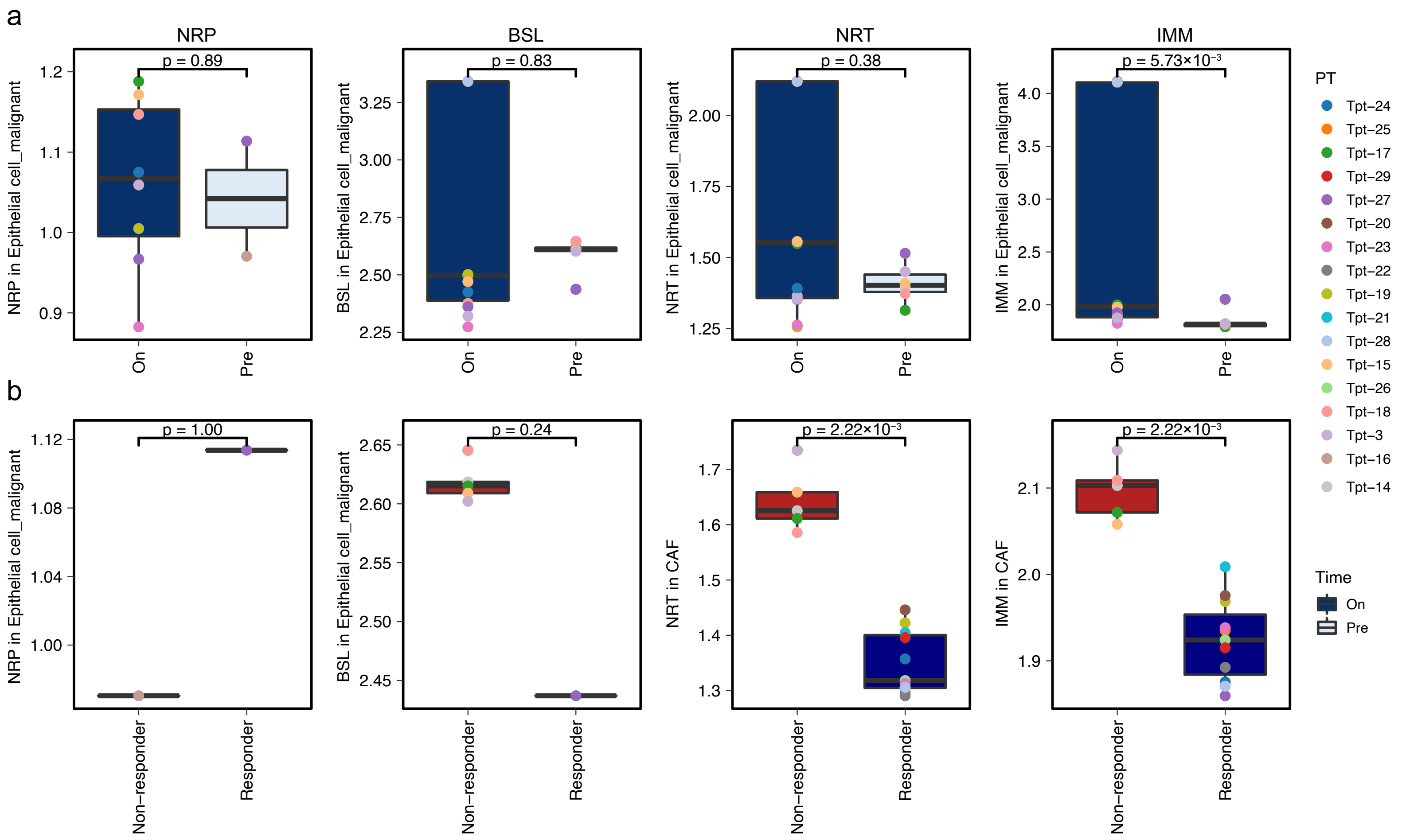

Supplementary Fig. 9. A comparison of the NRP program expression in malignant cells between different groups. a, Pre versus On. b, treated tumors from non-responders versus from responders. Boxplots indicated the median ± 1 quartile, with whiskers extending from the hinge to the smallest and largest values within 1.5 interquartile range from the box boundaries. Comparisons were performed by using the two-sided Wilcoxon rank-sum test.

Supplementary Fig. 10

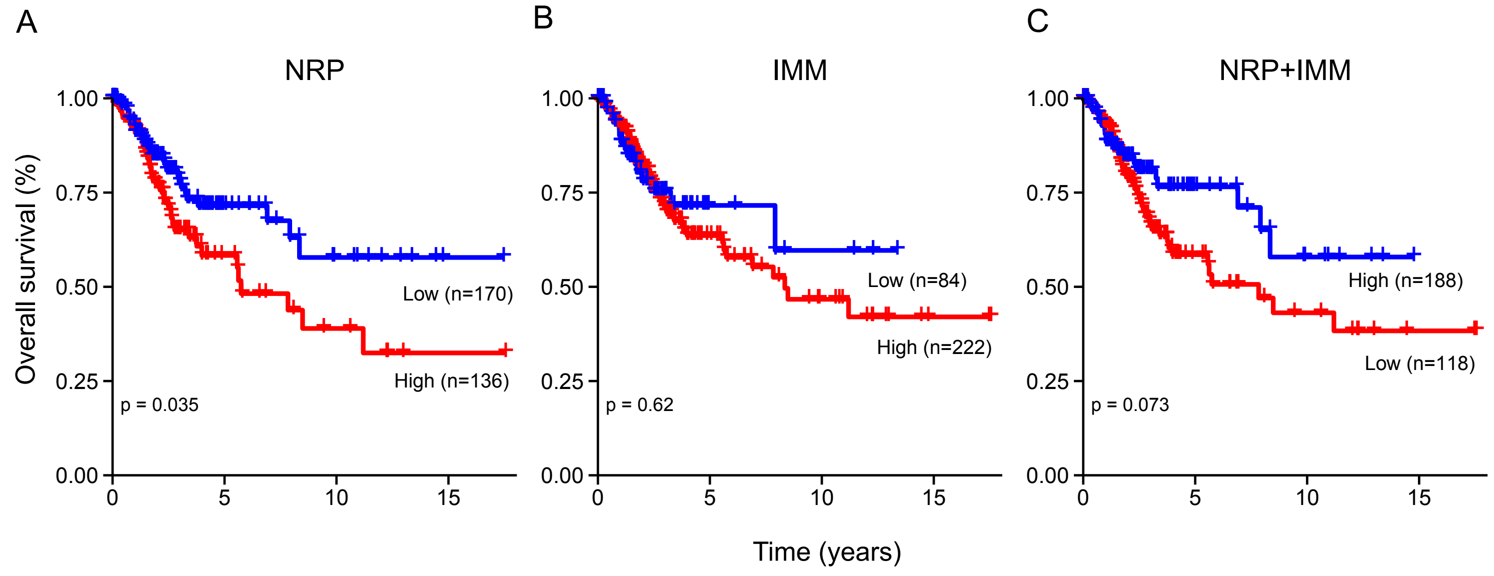

Supplementary Fig. 10. The overall survival of cases stratified by using the program expression levels. Statistical analysis was performed using Kaplan-Meier curves.

Supplementary Fig. 11

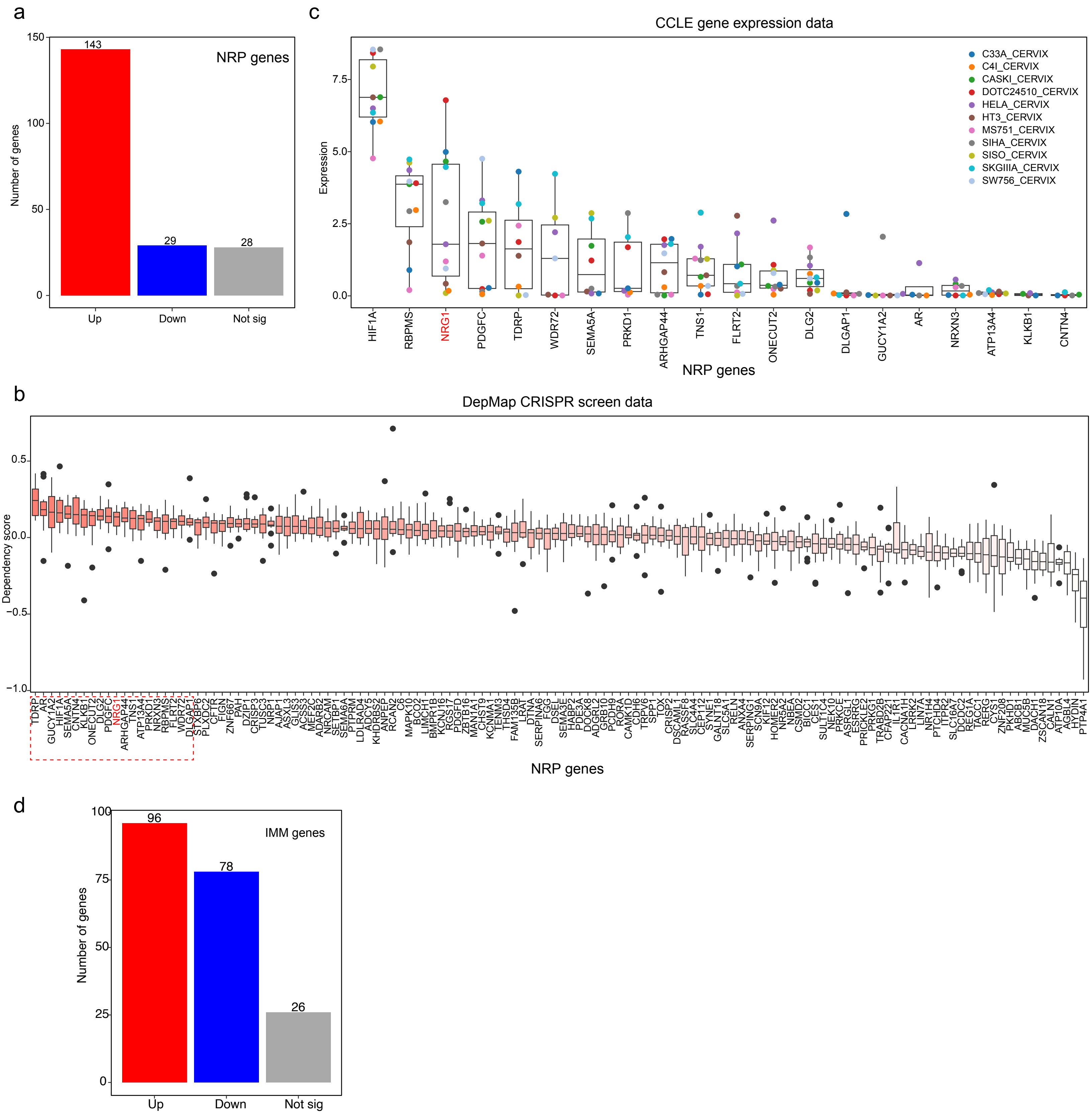

e

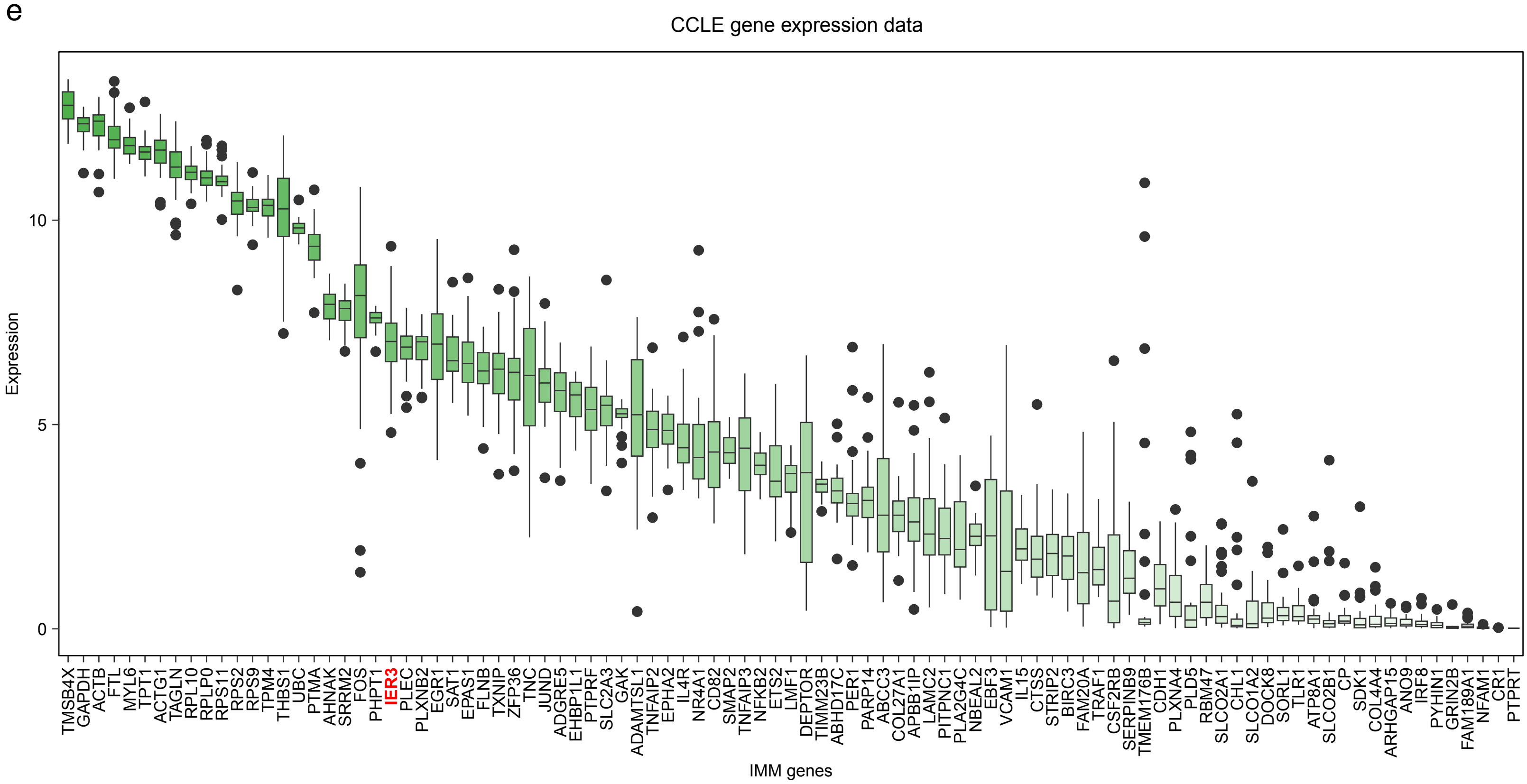

Supplementary Fig. 11. Expression of key NRP and IMM genes. (a) Number of differentially expressed NRP genes in malignant cells from non-responder versus responder after radiotherapy. (b) Boxplot of NRP gene dependency score of cervical cancer cell lines. (c) Endogenous gene expression levels of top 20 NRP genes obtained from gene dependency data in cervical cancer cell lines. The color of dots represents different cervical cell lines. (d) Number of differentially expressed IMM genes in malignant cells from non-responder versus responder after radiotherapy. (e) Endogenous gene expression levels of upregulated IMM genes in fibroblast cell lines. *P*-value was calculated by using the two-sided Wilcoxon rank-sum test. Boxplots indicate the median ± 1 quartile, with whiskers extending from the hinge to the smallest and largest values within 1.5 interquartile range from the box boundaries. FDR was calculated by using the Benjamini–Hochberg algorithm. Up: FDR < 0.05 and mean expression in malignant cells from non-responders higher than those from responders; Down: FDR < 0.05 and mean expression in malignant cells from non-responders lower than those from responders; Not sig: FDR > 0.05; CCLE: Cancer Cell Line Encyclopedia.

References

[1] B. Liang, Y. Huang, Y. Zhong, Z. Li, R. Ye, B. Wang, B. Zhang, H. Meng, X. Lin, J. Du, M. Hu, Q. Wu, H. Sui, X. Yang, Z. Huang, *J Hazard Mater* **2022**, *430*, 128459, https://doi.org/10.1016/j.jhazmat.2022.128459.

[2] R. Gao, C. Kim, E. Sei, T. Foukakis, N. Crosetto, L. K. Chan, M. Srinivasan, H. Zhang, F. Meric-Bernstam, N. Navin, *Nat Commun* **2017**, *8* (1), 228, https://doi.org/10.1038/s41467-017-00244-w.

[3] D. T. Le, J. N. Durham, K. N. Smith, H. Wang, B. R. Bartlett, L. K. Aulakh, S. Lu, H. Kemberling, C. Wilt, B. S. Luber, F. Wong, N. S. Azad, A. A. Rucki, D. Laheru, R. Donehower, A. Zaheer, G. A. Fisher, T. S. Crocenzi, J. J. Lee, T. F. Greten, A. G. Duffy, K. K. Ciombor, A. D. Eyring, B. H. Lam, A. Joe, S. P. Kang, M. Holdhoff, L. Danilova, L. Cope, C. Meyer, S. Zhou, R. M. Goldberg, D. K. Armstrong, K. M. Bever, A. N. Fader, J. Taube, F. Housseau, D. Spetzler, N. Xiao, D. M. Pardoll, N. Papadopoulos, K. W. Kinzler, J. R. Eshleman, B. Vogelstein, R. A. Anders, L. A. Diaz, Jr., *Science* **2017**, *357* (6349), 409, https://doi.org/10.1126/science.aan6733.

[4] J. C. M. Stephen J. Fleming, Mehrtash Babadi, *bioRxiv* **2019**, https://doi.org/10.1101/791699

[5] C. S. McGinnis, L. M. Murrow, Z. J. Gartner, *Cell Syst* **2019**, *8* (4), 329, https://doi.org/10.1016/j.cels.2019.03.003.

[6] I. Korsunsky, N. Millard, J. Fan, K. Slowikowski, F. Zhang, K. Wei, Y. Baglaenko, M. Brenner, P. R. Loh, S. Raychaudhuri, *Nat Methods* **2019**, *16* (12), 1289, https://doi.org/10.1038/s41592-019-0619-0.

[7] L. McInnes, J. Healy, J. Melville, UMAP: Uniform Manifold Approximation and Projection for Dimension Reduction. **2018**; p arXiv:1802.03426.

[8] D. Kim, B. Langmead, S. L. Salzberg, *Nat Methods* **2015**, *12* (4), 357, https://doi.org/10.1038/nmeth.3317.

[9] a) R. W. Yuanxing Wang, Shaojun Zhang, Shumei Song, Changying Jiang, Guangchun Han, Michael Wang, Jaffer Ajani, Andy Futreal, Linghua Wang, *bioRxiv* **2019**, https://doi.org/10.1101/507871; b) A. Sinjab, G. Han, W. Treekitkarnmongkol, K. Hara, P. M. Brennan, M. Dang, D. Hao, R. Wang, E. Dai, H. Dejima, J. Zhang, E. Bogatenkova, B. Sanchez-Espiridion, K. Chang, D. R. Little, S. Bazzi, L. M. Tran, K. Krysan, C. Behrens, D. Y. Duose, E. R. Parra, M. G. Raso, L. M. Solis, J. Fukuoka, J. Zhang, B. Sepesi, T. Cascone, L. A. Byers, D. L. Gibbons, J. Chen, S. J. Moghaddam, E. J. Ostrin, D. Rosen, J. V. Heymach, P. Scheet, S. M. Dubinett, J. Fujimoto, Wistuba, II, C. S. Stevenson, A. Spira, L. Wang, H. Kadara, *Cancer Discov* **2021**, *11* (10), 2506, https://doi.org/10.1158/2159-8290.CD-20-1285.

[10] A. M. Newman, C. B. Steen, C. L. Liu, A. J. Gentles, A. A. Chaudhuri, F. Scherer, M. S. Khodadoust, M. S. Esfahani, B. A. Luca, D. Steiner, M. Diehn, A. A. Alizadeh, *Nat Biotechnol* **2019**, *37* (7), 773, https://doi.org/10.1038/s41587-019-0114-2.

[11] L. Zhang, Y. Du, S. Xu, Y. Jiang, C. Yuan, L. Zhou, X. Ma, Y. Bai, J. Lu, J. Ma, *Cancer letters* **2019**, *442*, 242–251, https://doi.org/10.1016/j.canlet.2018.11.003.
